# Modeling ferroptosis in human dopaminergic neurons: Pitfalls and opportunities for neurodegeneration research

**DOI:** 10.1016/j.redox.2024.103165

**Published:** 2024-04-24

**Authors:** Nadine Renner, Franziska Schöb, Regina Pape, Ilinca Suciu, Anna-Sophie Spreng, Anna-Katharina Ückert, Eike Cöllen, Federica Bovio, Bruno Chilian, Johannes Bauer, Stefan Röpcke, Jörg Bergemann, Marcel Leist, Stefan Schildknecht

**Affiliations:** a Albstadt-Sigmaringen University, Faculty of Life Sciences, 72488, Sigmaringen, Germany; b In Vitro Toxicology and Biomedicine, Department of Biology, University of Konstanz, 78457, Konstanz, Germany; c Department of Biotechnology and Biosciences, University of Milano-Bicocca, 20126, Milano, Italy; d TRI Thinking Research Instruments GmbH, Große Freiheit 77, 22767, Hamburg, Germany; e Stemick GmbH, Byk-Gulden Str. 2, 78467, Konstanz, Germany

**Keywords:** Ferroptosis, LUHMES, Dopamine neurons, Hydroxyl radical, In vitro model, Parkinson's disease

## Abstract

The activation of ferroptosis is being pursued in cancer research as a strategy to target apoptosis-resistant cells. By contrast, in various diseases that affect the cardiovascular system, kidneys, liver, and central and peripheral nervous systems, attention is directed toward interventions that prevent ferroptotic cell death. Mechanistic insights into both research areas stem largely from studies using cellular *in vitro* models. However, intervention strategies that show promise in cellular test systems often fail in clinical trials, which raises concerns regarding the predictive validity of the utilized *in vitro* models.

In this study, the human LUHMES cell line, which serves as a model for human dopaminergic neurons, was used to characterize factors influencing the activation of ferroptosis. Erastin and RSL-3 induced cell death that was distinct from apoptosis. Parameters such as the differentiation state of LUHMES cells, cell density, and the number and timing of medium changes were identified as determinants of sensitivity to ferroptosis activation. In differentiated LUHMES cells, interventions at mechanistically divergent sites (iron chelation, coenzyme Q_10_, peroxidase mimics, or inhibition of 12/15-lipoxygenase) provide almost complete protection from ferroptosis. LUHMES cells allowed the experimental modulation of intracellular iron concentrations and demonstrated a correlation between intracellular iron levels, the rate of lipid peroxidation, as well as the sensitivity of the cells to ferroptotic cell death.

These findings underscore the importance of understanding the various factors that influence ferroptosis activation and highlight the need for well-characterized *in vitro* models to enhance the reliability and predictive value of observations in ferroptosis research, particularly when translating findings into *in vivo* contexts.

## Graphical abstract

**Figure:**
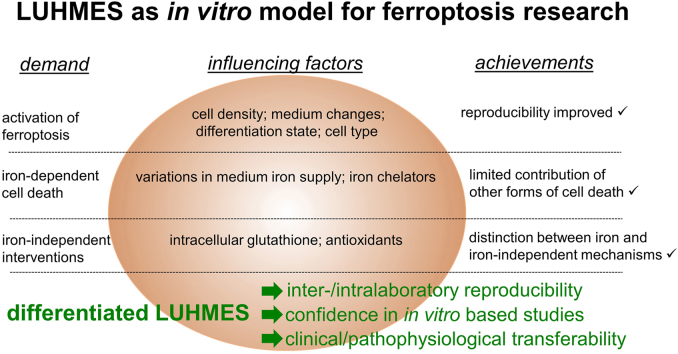

## 1 Introduction

Ferroptosis has been recognized as a form of regulated cell death contributing to the degeneration of nigrostriatal neurons associated with a Parkinsonian phenotype [1,2]. It involves iron-catalyzed formation of hydroxyl radicals and subsequent peroxidation of polyunsaturated fatty acids [3,4]. Accumulation of lipid peroxides alters the physical properties of lipid bilayers by reducing membrane fluidity, impairing lateral diffusion, or increasing membrane permeability, ultimately leading to cell damage and death [5]. Lipid peroxides can also originate from the activities of cellular enzymes such as lipoxygenases or members of the cytochrome P450 family [6,7]. Involvement of ferroptosis has been suggested for a variety of diseases affecting e.g. the kidney, liver, or the cardiovascular system [[8], [9], [10]]. However, Parkinsons disease-associated degeneration of dopamine neurons represents a prime example of ferroptosis activation, as nigrostriatal tissue is characterized by elevated total iron levels compared to other brain areas [11,12].

Within the cell, the cytosolic labile iron pool (LIP) reflects the share of “free” iron, capable of contributing to hydroxyl radical formation [13,14]. In addition to amino acids, ATP/ADP, and proteins, reduced glutathione (GSH) serves as a quantitatively relevant coordinator of cytosolic ferrous (Fe^2+^) iron [15]. To counteract the continuous formation of lipid peroxides, cells are equipped with a range of defense systems, including the selenoenzyme glutathione peroxidase 4 (GPx4), the ferroptosis suppressor protein 1 (FSP-1), or dihydroorotate dehydrogenase (DHODH) [[16], [17], [18]]. Ferroptosis is therefore regarded a consequence of an imbalance between the rate of lipid peroxidation and the cell's capacity to prevent or counteract it.

Despite some promising exceptions, such as the identification of hyperoxidized peroxiredoxin 3 [19], the inherent restriction in current ferroptosis research is still the lack of specific biomarkers that would allow a precise delineation of its contribution to pathological events. This situation becomes even more complicated as a growing body of evidence indicates that ferroptosis and apoptosis can occur either sequentially or cooperatively in cells [20,21]. Many *in vitro* models have been established to develop therapeutic approaches and better understand cell death mechanisms [[22], [23], [24]]. Similar to other modes of cell death, the results obtained in cell culture models of ferroptosis do not always translate into clinical situations. The main reasons for this lack of clinical translatability may be ***1)*** the high complexity and individual heterogeneity of processes determining tissue damage in humans, ***2)*** principal issues with cell culture quality and criteria to derive data from *in vitro* models [[25], [26], [27]], and ***3)*** the insufficient characterization of *in vitro* systems with respect to modeling a relevant disease process.

For the present study, the human LUHMES cell line was chosen as a representative model, as it not only exhibits cardinal features characteristic of ferroptosis but also allows the experimental induction of apoptosis as an alternative mode of cell death [28,29]. The LUHMES cell line was generated from primary human mesencephalic cells and allows the differentiation into post-mitotic neurons with a dopaminergic phenotype as an *in vitro* model for Parkinson's disease [[30], [31], [32]]. LUHMES cells exhibit autonomous pace-making activity and are consequently characterized by high metabolic turnover, contributing to their distinct responsiveness to the activation of ferroptosis [33,34]. The LUHMES model demonstrated stability in large-scale screenings [35,36] and a high degree of interlaboratory reproducibility [[37], [38], [39], [40]]. Therefore, this cell model was chosen as an example of how to document and verify unique, experimentally quantifiable, molecular key events that allow an unequivocal determination of ferroptotic cell death.

## 2 Materials and methods

### 2.1 Cell culture

LUHMES cells were maintained in proliferation medium, consisting of Advanced DMEM/F12 (Invitrogen), 1 × N2 (Invitrogen), l-glutamine (2 mM, Invitrogen), and recombinant basic FGF (40 ng/ml, R&D Systems). Differentiation was initiated by Advanced DMEM/F12 containing l-glutamine (2 mM), tetracycline (1 μg/ml, Sigma), and recombinant human GDNF (2 ng/ml, R&D Systems) for 2 days to ensure termination of proliferation. Pre-differentiated cells were then detached (trypsin 0.05 %, Gibco), counted, and seeded at a density of 30,000-90,000 cells/well into 96-well plates for additional 4 days (total differentiation period: 6 days) [[30], [31], [32]].

Astrocytes were differentiated from human stem cells as previously described [41]. For the co-culture experiments, astrocytes were added to day 2 predifferentiated LUHMES cells during seeding at an astrocyte:LUHMES cell ratio of 1:10. Cells were maintained for 4 additional days in LUHMES differentiation medium. QuinoMit Q_10_ was employed as Q_10_ source. QuinoMit Q_10_ and a liposome solution free of Q_10_ (background) was provided by MSE Pharmazeutika GmbH, Bad Homburg, Germany. Erastin and RSL-3 were obtained from Sigma/Merck. Stock solutions of 10 mM were prepared in DMSO. 10 x working solutions were prepared freshly in medium and added to the cells following intensive vortexing. It is important to note that both RSL-3 and erastin working solutions must be regarded as suspensions rather than solutions. Particular attention has to be spend on the way the working solution is added to the cells. Application to the cells needs to ensure that all cells of a well are exposed to the solution at the time of addition of erastin or RSL-3.

### 2.2 Cell viability

*Resazurin reduction assay*. Resazurin was added to the cells in 96 well plates for 60 min at a concentration of 5 μg/ml. Fluorescence (530 nm_ex_/590 nm_em_) was determined with a Tecan Infinite M200 reader. Cell viability was expressed as the percentage of fluorescence intensity of untreated controls. *Lactate dehydrogenase (LDH) release assay*. Supernatants were collected in separate 96 well plates. The corresponding cells were then lysed in phosphate-buffered saline/Triton X-100 (0.1 %) for at least 20 min. A volume of 20 μl of cell homogenate and its corresponding supernatant were individually analyzed for LDH activity by the addition of 180 μl reaction buffer, adjusted to pH 7.4 by titration of K_2_HPO_4_ (40 mM) and KH_2_PO_4_ (10 mM), supplemented with NADH (250 μM) and sodium pyruvate (700 μM). NADH consumption was followed at 340 nm at 1 min intervals for 10 min. Data were expressed as ΔOD_340_ (supernatant)/ΔOD_340_ (supernatant + cell homogenate).

### 2.3 Glutathione detection

Cells were lysed by 1 % sulfosalicylic acid (100 μl/well of 96 well plates). Cell lysate (20 μl) was diluted by the addition of 80 μl water. The reaction was initiated by the addition of 100 μl reaction buffer (100 mM sodium phosphate, pH 7.4), containing 5,5′-dithiobis (2-nitrobenzoic acid) (DTNB; 100 μM, Sigma), NADPH (200 μM, Roth), glutathione reductase (1 U/ml, Sigma), and EDTA (1 mM, Sigma). For normalization, total protein content was detected (ROTIQuant, Roth). If not otherwise indicated, the detection of glutathione included both reduced (GSH) and oxidized (GSSG) glutathione.

### 2.4 ATP detection

Cell Titer Glo 2.0 reagent (Promega) was mixed at a 1:1 ratio with 0.5 % Triton in PBS. Medium was removed. In the 96 well plate format, 100 μl of the mix was added and incubated for 10 min at 20 °C, followed by luminescence detection. The ATP standard curve covered a range from 0.15 μM to 10 μM.

### 2.5 Caspase-3 activity measurement

LUHMES cells grown in 96-well plates were lysed for 20 min at 20 °C in 50 μl of a hypotonic buffer composed of HEPES (25 mM, pH 7.4), MgCl_2_ (5 mM), EGTA (1 mM), Triton X-100 (0.1 %), and PEFA block (1 mM). Subsequently, 30 μl of the lysate was transferred to two individual 96-well plates. In one of these plates, the caspase-3 inhibitor Q-VD-OPH (1 μM, Cayman Chemicals) was added. Next, substrate buffer containing HEPES (50 mM, pH 7.4), sucrose (1 %), CHAPS (0.1 %), and DTT (10 mM) including Ac-DEVD-afc substrate (50 μM, AAT Bioquest) was added. Fluorescence intensity (excitation: 385 nm, emission: 505 nm) of free afc was measured at intervals of 20 min for 4 h. Background values obtained from samples treated with the caspase-3 inhibitor were subtracted to ensure accurate detection of caspase-3 activity. Human recombinant caspase-3 (Sigma) was used to generate calibration curves. Finally, values were normalized to the total protein content of the cells [42].

### 2.6 Colorimetric ferrozine-based iron detection

Intracellular iron (ferrous and ferric iron) was analyzed according to the protocol described by Riemer et al. [43]. Differentiated LUHMES in 10 cm dishes were washed twice with pre-warmed PBS. After removal of PBS, 200 μl NaOH (50 mM) was added, and cells were collected and transferred into a 1.5 ml tube. For complete lysis, cells were maintained on a shaker at 600 rpm at RT for 2 h. Then, 200 μl HCl (10 mM) was added. Each sample was sonicated on ice (Bandelin Sonopuls HD 3100 ultrasonic homogenizer, 70 % amplitude, 10 s pulses, 0.186 kJ/pulse) and subsequently supplemented with 200 μl of iron-releasing reagent (a freshly prepared solution of equal volumes of HCl [1.4 M] and KMnO_4_ [4.5 % w/v in water]) for 2 h at 60 °C and 600 rpm. Following cooling, 60 μl of iron detection reagent, consisting of ferrozine (6.5 mM), neocuproine (6.5 mM), ammonium acetate (2.5 M), and ascorbic acid (1 M), was added. The samples were incubated for 30 min at 600 rpm and centrifuged at 10,000×*g* for 3 min. A total of 280 μl supernatant was transferred to a 96-well plate, and absorbance was measured at 550 nm. Standard concentrations of FeCl_3_ (0–300 μM) were prepared in HCl (10 mM) and were treated exactly as the samples. The protein concentrations of the samples were determined using the ROTI Quant Universal Protein Assay Kit, and intracellular iron concentrations were normalized to the corresponding protein content.

### 2.7 Determination of lipid peroxidation

Lipid peroxidation was assessed using BODIPY™ 581/591 C11 (Invitrogen), which was added to the cell culture medium (96 well plate format) at a concentration of 2.5 μM. Following an incubation period of at least 20 min at 37 °C, the cells were detached using a 0.05 % trypsin solution and collected in PBS. The shift in fluorescence emission, indicative of BODIPY™ 581/591 C11 oxidation, was measured using flow cytometry (BD FACS Verse) with excitation at 488 nm. Specifically, the change from red fluorescence (detected using a 586 nm filter) to green fluorescence (detected using a 527 nm filter) was analyzed. A total of 10,000 cells were analyzed for each individual sample. Consistent gating parameters were applied within each experiment to distinguish between green and red populations.

### 2.8 Malondialdehyde assay

After the collection of cells (60 mm diameter dishes) in water, sonication on ice, and adjustment to equal protein content, a 100 μl sample was mixed with 200 μl of assay mix containing 15 % trichloroacetic acid and 0.375 % thiobarbituric acid in 250 mM HCl. Following thorough vortexing, the samples were boiled at 95 °C for 45 min and then centrifuged at 20,000×*g* for 10 min. The absorbance of the supernatant was detected at 535 nm and converted to equivalents of MDA using an absorbance coefficient of 1.56 × 10^5^ M^−1^ cm^−1^.

### 2.9 Immunocytochemistry

LUHMES monocultures were grown on 10 mm glass coverslips in 24 well plates (Menzel, Braunschweig, Germany), coated with poly-l-ornithine (0.1 mg/ml in PBS) at 37 °C for 1 h and washed three times with PBS. The glass coverslips were then coated with a mixture of fibronectin and laminin (1 μg/ml each) in PBS overnight. The coating solution was removed, and the cells were seeded without further washing. Following treatment, the cells were fixed with paraformaldehyde (4 %) for 15 min, permeabilized with PBS/Triton (0.1 %) for 20 min, blocked with bovine serum albumin (BSA, 5 %) for 1 h, and stained with an anti-β–III–tubulin antibody (rabbit, 1:500, Sigma T2200) in BSA (2.5 %) overnight at 4 °C. Following four washing steps with PBS, the secondary antibody (goat anti-rabbit; 1:250 in 2.5 % BSA; Kirkegaard and Perry No. 03-15-06) was added for at least 60 min. Glass coverslips were mounted on microscope slides with 10 μl of ROTImount (containing DAPI, Roth)

LUHMES/astrocyte co-cultures were fixed by replacing half the medium with 10 % neutral buffered formalin (Leica Biosystems Richmond, Inc., Richmond, IL, USA), incubated for 30 min at RT, and washed with Dulbecco's PBS without Ca^2+^ and Mg^2+^ (DPBS, Gibco by Thermo Fisher Scientific, Waltham, MA, USA). Cells were permeabilized in 0.6 % Triton-X100, Sigma) in DPBS for 10 min at RT. The permeabilization buffer was then replaced with 0.1 % Triton-X100 and 5 % fetal bovine serum in DPBS (blocking buffer) for 1 h at RT. Primary antibodies (anti-GFAP, Merck Millipore, AB5541; anti-TUJ1, BioLegend 801202) were diluted 1:1000 in blocking buffer, and cells were incubated with the antibodies at 4 °C overnight. The staining solution was removed, and the cells were gently washed three times with DPBS. Secondary antibodies (anti-chicken IgY, Alexa Fluor 555, Life Technologies A-21437; anti-mouse IgG2a, Alexa Fluor 488, Life Technologies A-21131) were diluted in blocking buffer, and H-33342 was added 1:1000. Cells were incubated with the secondary antibodies for 1 h at RT, washed three times with DPBS, and stored in DPBS at 4 °C. Imaging was performed using a Zeiss AxioObserver epifluorescence microscope with ZEN 2 Pro Blue Edition software (Zeiss, Oberkochen, Germany), and images were processed in ImageJ (FIJI version 1.51s).

### 2.10 Label-free quantification of neurite mass

Fixed cells in 96 well plates (paraformaldehyde 4 % for 15 min) were imaged with a resolution of 1280 × 1024 pixels at 2 px/μm (40 × ) using the VAIDR system [44]. Z-stacks were recorded automatically at nine positions per well on a regular grid of 3 × 3 positions. If focusing failed, the respective stacks were automatically excluded from the analysis. Thirteen random images were labeled manually using the VAIDR labeling user interface. The system was trained to distinguish between areas depicting neurites, somata, and background. A U-Net++-based artificial neural network (implemented in Keras with TensorFlow) was trained to replicate hand labeling on the remaining images [45]. To do this, the system automatically reduced labeled z-stacks to three planes: the sharpest plane, the one 4 μm above it, and the one 4 μm below it. The system normalized the intensities locally using a Gaussian kernel and segmented the three plane stacks and corresponding stack labels into cutouts of 128 × 128 pixels each. The network was trained on segmented images and labels.

All stacks were scored, and relative areas were generated by selecting, for each pixel position in each stack, the class (neurite, soma, or background) that had received the highest model score. Area ratios were computed per stack, and the median was computed for each well.

### 2.11 Mitochondrial respiration

LUHMES cells at day 2 of differentiation were seeded into PLO/fibronectin-coated “Seahorse 96-well plates” (Agilent, Santa Clara, CA, USA) at a density of 30,000 cells/well. On day 5 of differentiation, a partial medium change was conducted (50 %), and on day 6, cells were exposed to ferroptosis activators. For detection of respiration, the medium was replaced with Agilent Seahorse XF DMEM (pH 7.4), containing 18 mM glucose, 2 mM glutamine, and 1 mM pyruvate. The Mito Stress Test was performed by sequential injection of oligomycin (1 μM), carbonyl cyanide-4-(trifluoromethoxy) phenylhydrazone (FCCP; 1.5 μM), and rotenone/antimycin A (0.5 μM each). The total number of cells in each well was quantified for normalization of the oxygen consumption rate.

### 2.12 Sample preparation for the transcriptomics experiment

Differentiated (day 6) LUHMES cells in 96-well plates (60,000 cells/well) were treated with erastin, RSL-3, or cadmium. For sample preparation, the medium was replaced by Biospyder Lysis Buffer (33 μl/well; Biospyder Tech., Glasgow, UK) for 15 min, the plates were then sealed and frozen at −80 °C. Targeted transcriptome sequencing (including QC, alignment, and read quantification) was conducted at Bioclavis (Biospyder Tech.) using TempO-Seq technology. For each of the 3257 targeted genes, a 50 bp fragment was amplified, and sample-specific barcodes were introduced to allow sample pooling for next-generation sequencing. A reference library containing a collection of all amplification products was used to assign read counts to each target gene. A pre-filtering step for the library size (<0.2 million) and average gene count (<1.5) was performed. Differential gene expression analysis of each treatment against the control group was conducted using the Wald test implemented in DESeq2/R, including an FDR correction using the Benjamini–Hochberg algorithm. To check for functional enrichment, we applied the WMEAN algorithm from decoupleR to gene expression statistics (stat) mapped onto the DoRothEA regulons and PROGENy pathway signatures.

### 2.13 Statistics

Data are presented as mean ± SD of biological triplicates. Each biological replicate included a variable number of technical replicates. The normal distribution of the data was assessed using the Shapiro-Wilk test. Given the low number of replicates (biological triplicates; technical replicates between 4 and 8), the data are considered to be normally distributed and of equal variance across samples. Individual outlieres were carfully investigated and measurements repeated if necessary. Differences were tested for significance either by one-way ANOVA followed by Dunnet's post hoc test, or by two-way ANOVA, followed by Bonferroni's multiple comparison post hoc test. Unless otherwise indicated, statistical significance was defined as *p* < 0.05. Statistical analysis was performed using GraphPad Prism 5.0 (GraphPad Software, La Jolla, USA).

## 3 Results

### 3.1 Activation of ferroptosis

**Fig. 1:**
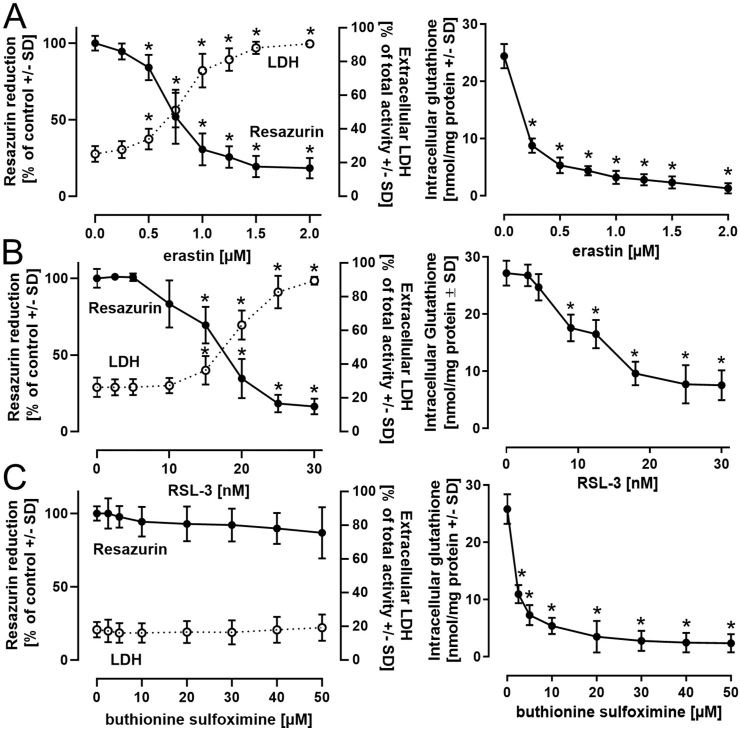
**Ferroptosis induction by erastin and RSL-3 in the LUHMES model. *A-B****)* LUHMES seeded at a density of 240,000 cells/cm^2^ were differentiated for 6 days and treated with the ferroptosis activators erastin (24 h) ***(A)*** or with RSL-3 (18 h) ***(B)***. Cell viability was assessed by resazurin reduction and LDH release assays. In parallel, the total content of intracellular glutathione (reduced and oxidized) was detected. ***C)*** As a reference for the role of glutathione in the observed cell death, buthionine sulfoximine, a γ-glutamyl-cysteine synthase inhibitor, was administered for 24 h. Data are presented as mean ± SD of biological triplicates. Each biological replicate included 6 technical replicates. The normal distribution of the data was assessed using the Shapiro-Wilk test. Differences were tested for significance by one-way ANOVA, followed by Dunnett's multiple comparison post hoc test. *p < 0.05 for comparison with untreated controls. Statistical analysis was performed using GraphPad Prism 5.0 (GraphPad Software, La Jolla, USA).

In the present study, the human neuronal LUHMES cell line was selected as an experimental *in vitro* model to characterize a set of molecular events associated with ferroptotic cell death. Ferroptosis was triggered in differentiated (day 6) LUHMES cells by the cystine/glutamate antiporter (system X_c_^−^) inhibitor erastin (Fig. 1A) or RSL-3 (Fig. 1B). An adverse effect on cell viability by erastin concentrations of approximately 1 μM and RSL-3 concentrations of approximately 20 nM was observed and is corroborated by similar findings in the literature [28,29]. To investigate whether cellular glutathione depletion is sufficient for ferroptosis activation, the γ-glutamyl-cysteine synthase inhibitor buthionine sulfoximine (BSO) was applied and caused an expected drop in intracellular glutathione concentration, equivalent to the effect evoked by erastin (Fig. 1C). In contrast to erastin, cell viability was not affected by BSO under the conditions employed (after a prolonged incubation for 48 h, a negative influence on viability was observed), indicating that intracellular glutathione depletion alone is insufficient to induce ferroptosis (Fig. 1C). This notion was supported by investigations of the time- and concentration-dependent effects of erastin and RSL-3 on viability and glutathione content of LUHMES cells (Suppl. Fig. 1). While the observed drop in intracellular glutathione evoked by RSL-3 largely followed the loss in cell viability, even the lowest erastin concentration applied (0.25 μM) resulted in maximal impairment of intracellular glutathione levels, despite a concentration-dependent influence on cell viability. These observations indicate that glutathione cannot be regarded as an independent factor for determining ferroptotic cell death.

### 3.2 Discrimination between ferroptosis and other forms of cell death

**Fig. 2:**
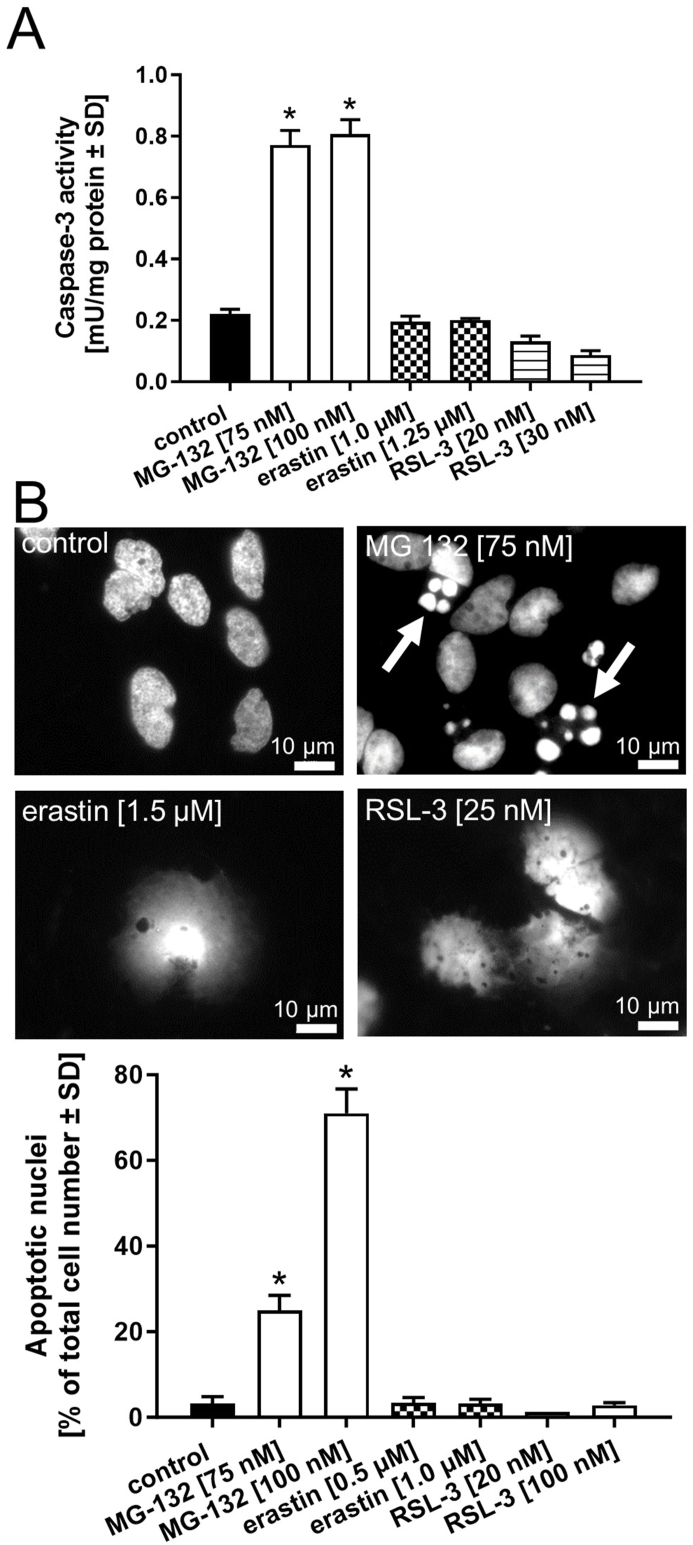
**Delineation of ferroptosis from apoptosis. *A****)* As a cardinal marker of apoptosis, activation of caspase-3 was determined in LUHMES cells (day 6) exposed to the apoptosis inducer MG-132 (an inhibitor of the proteasomal system) or the ferroptosis activators erastin or RSL-3 for 16 h in biological triplicates. ***B)*** Apoptotic nuclei were visualized with H-33342, assessed as an alternative marker of apoptosis, following treatment of the cells with erastin, RSL-3, or MG-132. For each condition, 500 nuclei were analyzed according to their size and morphology. Data are presented as mean ± SD of biological triplicates. Each biological replicate included 4 technical replicates. The normal distribution of the data was assessed using the Shapiro-Wilk test. Differences were tested for significance by one-way ANOVA, followed by Dunnett's multiple comparison post hoc test. *p < 0.05 for comparison with untreated controls. Statistical analysis was performed using GraphPad Prism 5.0 (GraphPad Software, La Jolla, USA).

Ferroptosis is characterized by increased iron-catalyzed formation of free radicals. Oxidative stress, however, is also a characteristic feature of apoptosis [46]. In the absence of robust and unambiguous molecular markers of ferroptosis, the experimental assessment of well-established key apoptotic events is suggested as an indirect strategy to exclude considerable involvement of apoptosis. The activation of caspase-3 is a cardinal molecular event of apoptotic death in LUHMES cells when exposed to the apoptosis inducer MG-132 (an inhibitor of the proteasomal system) but not when treated with erastin or RSL-3 (Fig. 2A). As an independent marker of apoptosis, the morphology of cell nuclei was also assessed (Fig. 2B). For quantification, 500 nuclei per condition were analyzed, apoptotic nuclei were determined according to a defined set of evaluation criteria. As hypothesized, MG-132 evoked not only the activation of caspase-3 but also the appearance of apoptotic nuclei. However, this was not observed following erastin or RSL-3 treatment, indicating the absence of a substantial contribution of apoptosis to the cell death observed following erastin or RSL-3 administration. Interestingly, both erastin and RSL-3 treatment of differentiated LUHMES cells revealed enlarged, disintegrated nuclei potentially evoked by oxidative damage to nuclear membranes. Whether this observation is a LUHMES-specific phenomenon or of general relevance to ferroptotic cell death needs to be determined.

### 3.3 Application of iron chelators to prevent ferroptosis

**Fig. 3:**
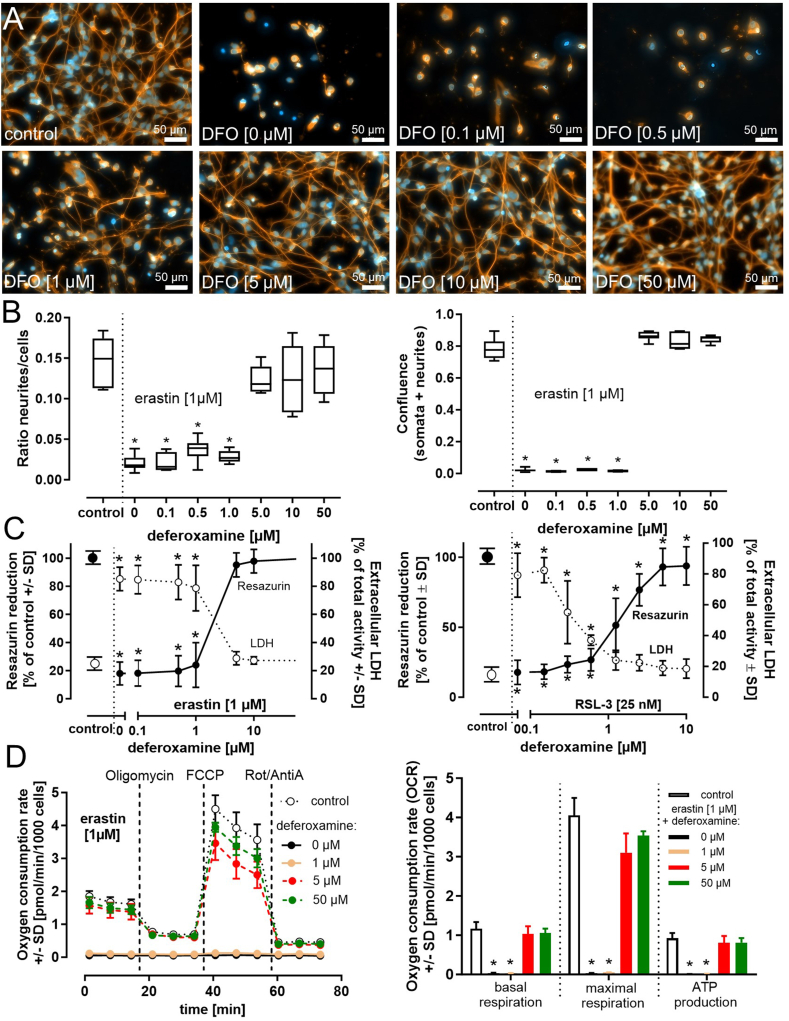
**Iron chelation protects LUHMES cells from ferroptosis**. Differentiated (day 6) LUHMES cells (240,000 cells/cm^2^) in regular, iron-containing Advanced DMEM/F12 medium, were pre-treated with the iron chelator deferoxamine (DFO) for 30 min, followed by the exposure to erastin (1 μM) for 24 h. ***A)*** For visualization of LUHMES cell morphology, cells were stained with an anti-β–III–tubulin antibody (orange). Nuclei were visualized with H-33342 (blue). ***B)*** Neurite integrity was quantified under the same conditions by automated, label-free microscopy. This algorithm allowed a discrimination between neurites and cell bodies and quantifies the area covered by neurites or cell bodies. ***C)*** Viability was determined by the analysis of resazurin reduction and LDH release. Erastin was added for 24 h, RSL-3 for 18 h. ***D)*** Changes in mitochondrial respiration were analyzed by the detection of the oxygen consumption rate (OCR) within individual wells of a 96-well plate. LUHMES cells (day 6) were exposed to erastin (1 μM) for 24 h in the presence of various deferoxamine concentrations as indicated. The OCR was normalized to the cell number. The mitochondrial complex V inhibitor oligomycin evokes an indirect inhibition of mitochondrial electron flow and allows a quantitative assessment of mitochondrial ATP (ATP production). The uncoupler FCCP is added to allow maximal electron flow (maximal respiration). Rotenone (Rot)/antimycin A (AntiA) block the mitochondrial respiratory chain and allow the assessment of non-mitochondrial respiration (basal respiration). Data (in ***B-D***) are presented as mean ± SD of at least 3 biological replicates. Each biological replicate included 6 (***B-D***) technical replicates. The normal distribution of the data was assessed using the Shapiro-Wilk test. Differences were tested for significance by one-way ANOVA, followed by Dunnett's multiple comparison post hoc test. *p < 0.05 for comparison with untreated controls. Statistical analysis was performed using GraphPad Prism 5.0 (GraphPad Software, La Jolla, USA). (For interpretation of the references to color in this figure legend, the reader is referred to the Web version of this article.)

Considering the iron-catalyzed formation of hydroxyl radicals in ferroptosis, the pharmacological decrease of the pool of intracellular redox-active iron by iron chelators appears to be an evident strategy to counteract ferroptotic cell death. To investigate the potential protective role of iron chelators, differentiated (day 6) LUHMES cells were treated with varying concentrations of the iron chelator deferoxamine and 1 μM erastin, and the cell morphology was visualized using β–III–tubulin staining (Fig. 3A). Neurite mass was quantified using a label-free automated system (Fig. 3B). The data indicated an erastin-dependent loss of both neurites (Fig. 3A + B) and cell viability (Fig. 3C), which was prevented in a concentration-dependent manner by deferoxamine (Fig. 3A–C). These observations were further supported by the findings illustrated in Suppl. Fig. 1D confirming an erastin or RSL-3 concentration-dependent loss of neurite mass accompanying the loss of viability. The literature reports alterations of mitochondrial morphology and function during ferroptosis [24,47,48]. Mitochondrial respiration was therefore assessed as a readout for the experimental quantification of mitochondrial (dys)function. Erastin (or RSL-3; data not shown) evoked a complete loss of mitochondrial basal respiration, ATP production, and ionophore-triggered maximal respiration. Deferoxamine allowed, in a concentration-dependent manner, the complete protection of basal respiration and ATP production and partially protected maximal respiration (Fig. 3D and Suppl. Fig. 2).

### 3.4 Iron-independent pharmacological interventions

**Fig. 4:**
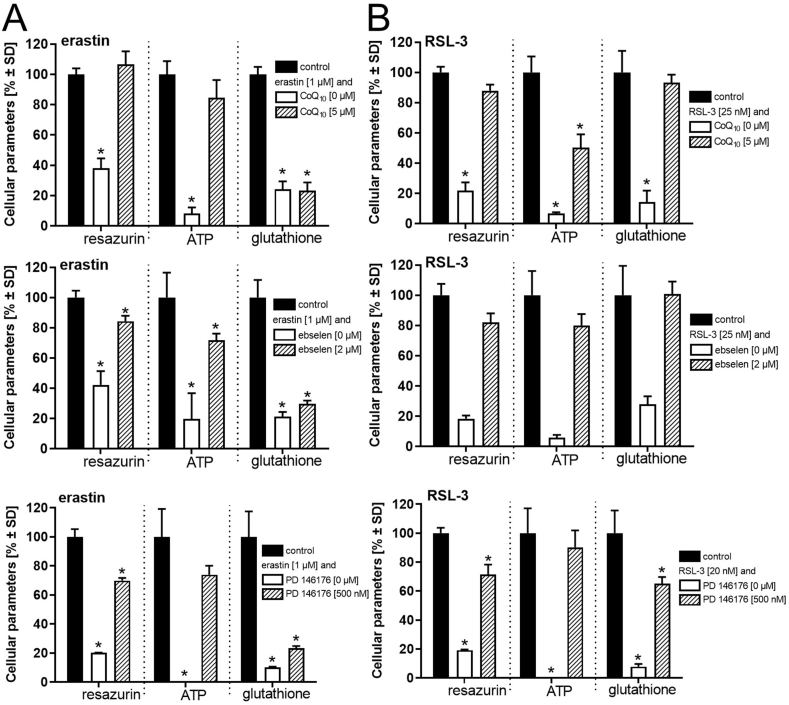
**Protection from ferroptosis**. LUHMES cells were pre-treated with coenzyme Q_10_ (CoQ_10_), the glutathione peroxidase mimic ebselen, or the 15-lipoxygenase inhibitor PD 146176 for 30 min, followed by incubation with ***A)*** erastin (1 μM) for 24 h or with ***B)*** RSL-3 (25 nM) for 18 h. The influence of CoQ_10_, ebselen, and PD 146176 on cell viability and intracellular glutathione and ATP concentrations was evaluated. Data are presented as mean ± SD of biological triplicates. Each biological replicate included 6 technical replicates. The normal distribution of the data was assessed using the Shapiro-Wilk test. Differences were tested for significance by one-way ANOVA, followed by Dunnett's multiple comparison post hoc test. *p < 0.05 for comparison with untreated controls. Statistical analysis was performed using GraphPad Prism 5.0 (GraphPad Software, La Jolla, USA).

For the rigorous characterization of an experimental *in vitro* cell model, intervention at mechanistically different sites, as elaborated in the literature, is an indispensable step in demonstrating its robustness and representation of ferroptosis. Differentiated LUHMES cells were treated with the membrane-targeted antioxidant coenzyme Q_10_, the glutathione peroxidase mimic ebselen, or the 15-lipoxygenase inhibitor PD 146176 (Fig. 4). The activity of lipoxygenases contributes to the enzymatic formation of lipid peroxides, coenzyme Q_10_ protects from lipid peroxidation when present in plasma or mitochondrial membranes, and ebselen acts as an antioxidant and a glutathione peroxidase mimic. These independent pharmacological interventions individually allowed partial or even complete protection from RSL-3-induced decreases in viability, ATP levels, and glutathione concentrations (Fig. 4B). All of these interventions protected from the loss of viability and intracellular ATP, but not glutathione, induced by erastin (Fig. 4A). These findings indicate that interference at only one of these individual key mechanistic events of ferroptosis was sufficient to achieve almost complete protection from erastin- or RSL-3-evoked cell death.

### 3.5 Influence of the experimental setup on ferroptosis induction

**Fig. 5:**
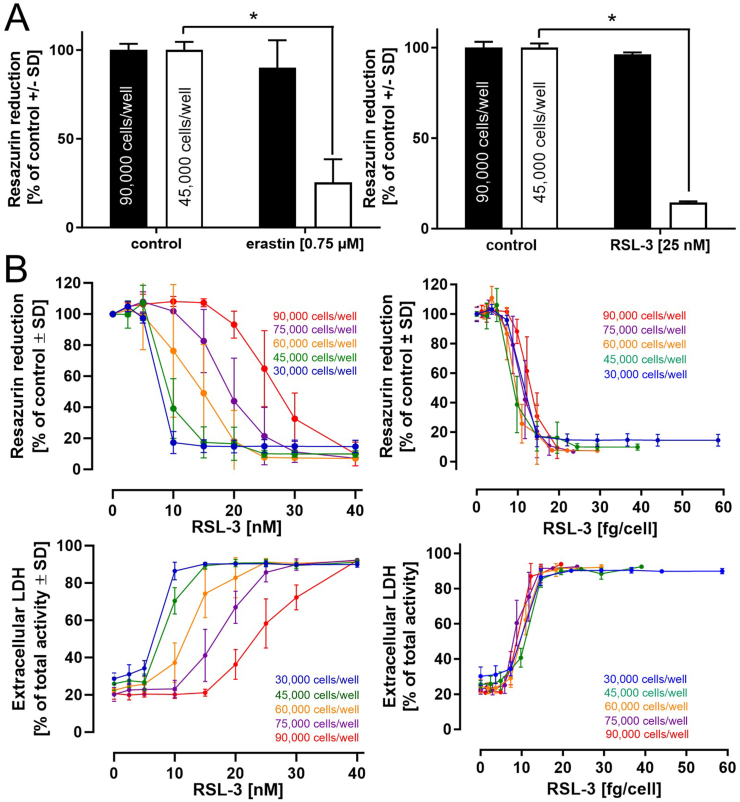
**Influence of cell density on ferroptosis activation**. After a 2-day predifferentiation period, LUHMES cells were seeded at a density of 90,000 or 45,000 cells/well (96 well plate; corresponds to 300,000 or 150,000 cells/cm^2^). At day 6 of differentiation, the cells were treated with ***A)*** erastin (24 h, 0.75 μM) or RSL-3 (18 h, 25 nM). For the detection of cell integrity, resazurin reduction, LDH release, and intracellular glutathione concentration were determined. ***B)*** LUHMES, seeded at the densities indicated, were exposed to varying concentrations of RSL-3 for a period of 18 h. Loss of viability is expressed depending on the concentration of RSL-3 (left). The same data sets are illustrated as exposure of an individual cell towards the corresponding doses of RSL-3 (right; in femtogram per cell). Data (in ***A*** and ***B***) are presented as mean ± SD of biological triplicates. Each biological replicate included 3 technical replicates. The normal distribution of the data (in ***A***) was assessed using the Shapiro-Wilk test. Differences were tested for significance by one-way ANOVA, followed by Dunnett's multiple comparison post hoc test. *p < 0.05 for comparison with untreated controls. Statistical analysis was performed using GraphPad Prism 5.0 (GraphPad Software, La Jolla, USA).

**Fig. 6:**
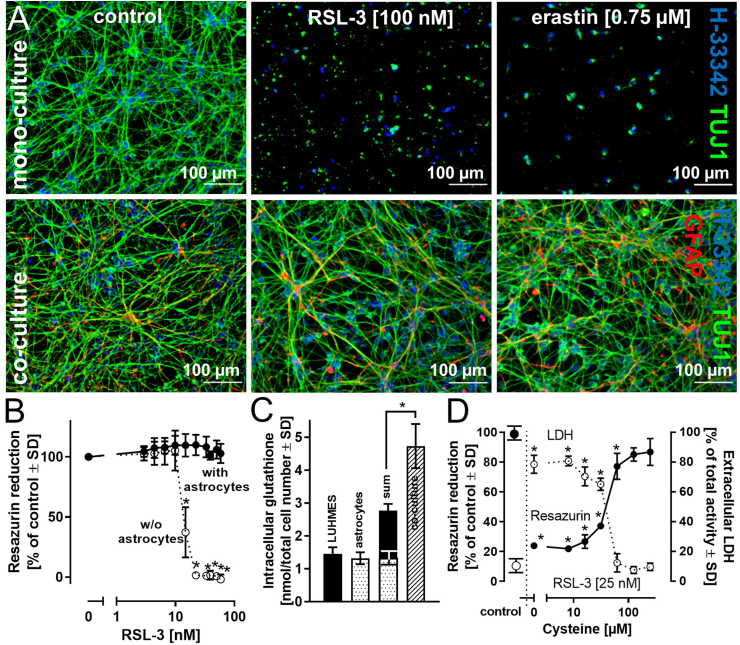
**Protection from ferroptosis by modulation of glutathione availability**. *A)* LUHMES-astrocyte co-culture. LUHMES cells were either differentiated as monocultures or at a ratio of 90 % LUHMES cells and 10 % astrocytes to achieve a total cell number that is equivalent to that in the monocultures. At day 6 of differentiation, cells were exposed to RSL-3 (18 h) or erastin (24 h). For visualization of LUHMES morphology, fixed cells were stained with an anti-β–III–tubulin antibody, and astrocytes were stained with an anti-GFAP antibody. Nuclei were visualized with H-33342. ***B)*** LUHMES cells in mono- or co-cultures were exposed to RSL-3 at different concentrations for 18 h. Cell viability was assessed using the resazurin reduction assay. ***C)*** Intracellular glutathione concentrations were determined in monocultures of LUHMES cells or astrocytes. The cell number was identical to that in the co-culture. In addition, intracellular glutathione concentrations were measured in the co-culture. ***D)*** To assess the influence of added cysteine on ferroptotic cell death, LUHMES cells in monoculture were treated with varying concentrations of cysteine together with RSL-3 for 18 h. Viability was determined using resazurin reduction and LDH release assays. Data are presented as mean ± SD of biological triplicates. Each biological replicate included 8 technical replicates. The normal distribution of the data was assessed using the Shapiro-Wilk test. Differences in ***B)*** between LUHMES mono- and co-cultures were tested for significance by two-way ANOVA, followed by Bonferroni's post hoc test; differences in ***C) + D)*** were tested for significance by one-way ANOVA, followed by Dunnett's post hoc test. *p < 0.05. Statistical analysis was performed using GraphPad Prism 5.0 (GraphPad Software, La Jolla, USA).

The experimental setup can have a profound influence on the outcomes of *in vitro* studies. Three exemplary parameters were selected to draw attention to their potential influence on ferroptosis in this *in vitro* cell model. ***1) Cell density***. To investigate the influence of cell density on the induction of ferroptosis, predifferentiated (nonproliferating) LUHMES cells on day 2 were seeded at a density of 150,000 or 300,000 cells/cm^2^ (=45,000 and 90,000 cells/well of a 96 well plate) and differentiated until day 6. Analysis of cell viability and cellular glutathione levels upon exposure to erastin or RSL-3 indicated pronounced cell death at a density of 150,000 cells/cm^2^, but little or no response was observed when the cell number was doubled (Fig. 5A and Suppl. Fig. 3). These observations underline the necessity for a precisely defined ratio between cell mass/volume and erastin or RSL-3 concentrations. LUHMES were therefore seeded at five different densities and incubated with different concentrations of RSL-3 for 18 h. Cell viability in a first step was illustrated for each individual cell density in a RSL-3 concentration-dependent manner and indicates a more pronounced activation of cell death at lower cell densities at a given RSL-3 concentration (Fig. 5B left). The same viability data were then illustrated in a dose-dependent manner (in femtogram per cell). This depiction of the data clearly indicates a distinct dose-dependency in the activation of ferroptosis by RSL-3 (and erastin) (Fig. 5B right). ***2) Influence of the cell model.*** To elucidate the influence of the cell model on ferroptosis activation, proliferating and differentiated (day 6) LUHMES cells were compared regarding their responses to the ferroptosis activators erastin and RSL-3. Cell numbers were adjusted to ensure identical cell counts at the time of treatment with erastin or RSL-3. In proliferating LUHMES cells, neither compound significantly influenced cell viability (Suppl. Fig. 4A and B) at erastin or RSL-3 concentrations that led to the complete loss of viability in differentiated LUHMES cells. The human hepatocarcinoma cell line HepG2 was used as an alternative cell model. In HepG2 cells, erastin evoked a decrease in intracellular glutathione levels at concentrations comparable to those that caused a decline in intracellular glutathione concentrations in differentiated LUHMES cells. However, no adverse effects on cell viability were detected in HepG2 cells (Suppl. Fig. 4C). Similarly, treatment of HepG2 or the mouse macrophage line J774A.1 with RSL-3 up to 1000 nM showed no influence on cell viability (Suppl. Fig. 4D). ***3) Influence of other cell types on the activation of ferroptosis.*** Mutual interactions between different cell types in tissues and organs can profoundly influence cell function and viability. Regarding dopaminergic neurons, the literature indicates several interactions with astrocytes, affecting neuronal energy metabolism, iron handling and storage, and the supply of neurons with glutathione, among others [49]. To illustrate the relevance of such interactions to neuronal survival under pro-ferroptotic conditions, LUHMES cells were differentiated either in monoculture or in co-culture with human stem cell-derived astrocytes at a LUHMES cell/astrocyte ratio of 10:1. Erastin and RSL-3 induced a concentration-dependent decline in LUHMES viability when present as monoculture. However, the presence of astrocytes resulted in complete protection from cell death under similar conditions (Fig. 6A+B). Among the potential protective mechanisms involved, we focused on glutathione as a potential determinant of cell death (Suppl. Fig. 5A). In the presence of astrocytes, intracellular glutathione levels were significantly higher in neurons in the co-culture system than in those in monocultures. The total amount of glutathione in the co-culture even exceeded the sum of the glutathione levels detected in LUHMES cells and astrocytes in separate monocultures (Fig. 6C). The application of cysteine (Fig. 6D) or GSH (Suppl. Fig. 5B) to a LUHMES monoculture almost doubled the amount of intracellular glutathione, culminating in elevated resistance against ferroptosis induction (Fig. 6D + Suppl. Fig. 5C).

### 3.6 The role of iron

**Fig. 7:**
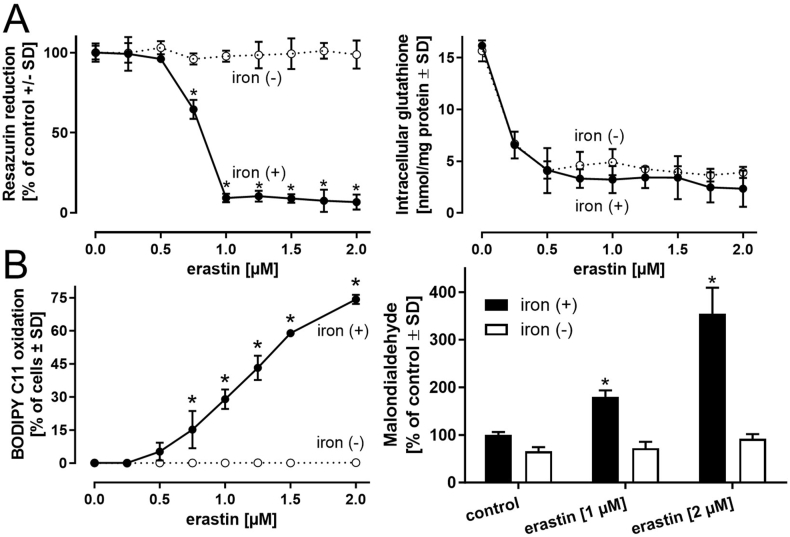
**Influence of iron availability on ferroptosis activation**. LUHMES cells were differentiated for 2 days in regular medium and were then seeded either in regular iron-containing differentiation medium (iron (+)), or in medium devoid (iron (−)) of the iron components ferric nitrate (0.05 mg/l), ferric sulfate (0.417 mg/l), and human holo-transferrin (7.5 mg/l) present in Advanced DMEM/F12 medium. Iron-free medium was prepared according to the composition of Advanced DMEM/F12 provided by Gibco/Thermo Fisher. After 4 additional days of differentiation (i.e., a total of 6 days), the cells were treated with different concentrations of ***A)*** erastin for 24 h. Viability was assessed by measuring the reduction of resazurin, and the glutathione concentration (reduced and oxidized) was determined. ***B)*** Lipid peroxidation was determined by the fluorescent probe sensor BODIPY™ 581/591 C11. Alternatively, malondialdehyde, as a product of lipid peroxidation, was detected. Data are presented as mean ± SD of biological triplicates. Each biological replicate included 6 technical replicates. The normal distribution of the data was assessed using the Shapiro-Wilk test. Differences between iron (+) and iron (−) cells exposed to erastin or RSL-3 were tested for significance by two-way ANOVA, followed by Bonferroni's post hoc test. In the malondialdehyde assay, statistical difference between erastin treatment and untreated controls was tested by one-way ANOVA, followed by Dunnett's post hoc test. *p < 0.05. Statistical analysis was performed using GraphPad Prism 5.0 (GraphPad Software, La Jolla, USA).

To characterize the influence of extracellular iron supply on the tendency of LUHMES cells to undergo ferroptotic cell death, LUHMES cells were differentiated from day 2 to day 6, either in the presence (iron (+)) or absence (iron (−)) of iron, and then exposed to varying concentrations of erastin or RSL-3 (Fig. 7A and Suppl. Fig. 6). Iron (−) medium had no influence on the observed erastin-mediated decrease in glutathione levels but prevented the loss of cell viability and ATP (Fig. 7A and Suppl. Fig. 6). Differentiation in iron (−) medium revealed the complete protection of viability, glutathione, and ATP from RSL-3-dependent deterioration (Suppl. Fig. 6). In iron (+) cells, lipid peroxidation was assessed by BODIPY™ 581/591 C11 oxidation and correlated with the decline in cell viability (Fig. 7B and Suppl. Fig. 6C). In iron (−) cells, no significant BODIPY™ 581/591 C11 oxidation could be detected (Fig. 7 B). As alternative readout, the formation of malondialdehyde was assessed and provided qualitatively similar results (Fig. 7B).

**Fig. 8:**
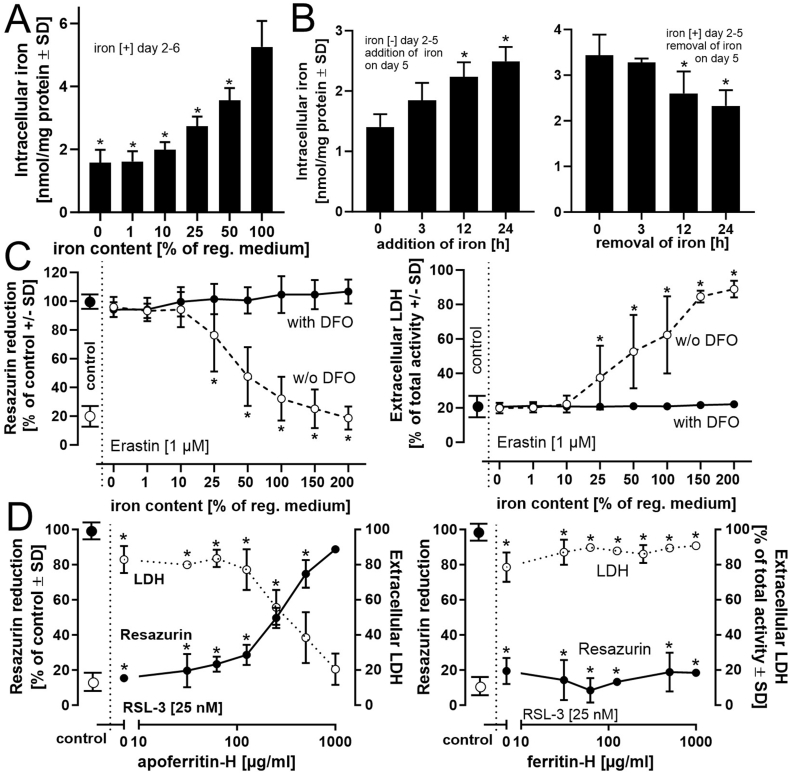
**Modulation of intracellular iron levels influences ferroptosis induction. *A****)* Detection of intracellular iron content. LUHMES cells were differentiated (days 2–6) in the presence of various amounts of total iron supply present in regular medium. Intracellular iron was determined by a ferrozine-based colorimetric assay. The value of 100 % reflects the iron supply representative of regular Advanced DMEM/F12 medium (ferric nitrate 0.05 mg/l; ferric sulfate 0.417 mg/l; human holo-transferrin 7.5 mg/l). ***B)*** The influence of iron depletion/addition in the medium on intracellular total iron content was investigated on day 5 in LUHMES cells kept on days 2–5 in regular medium (iron [+]) or iron-free medium (iron [−]). At day 5, the medium was changed from iron [−] to iron [+] or vice versa for 3, 12, or 24 h, followed by the analysis of intracellular iron concentrations. ***C)*** LUHMES, differentiated (day 2–6) in the presence of different percentages of iron supply (100 % eqials iron content in regular medium) were exposed to the iron chelator deferoxamine (DFO) (50 μM) and erastin (1 μM) for 24 h. Viability was determined by the resazurin reduction and the LDH release assays. ***D)*** Influence of the iron-storage protein ferritin on ferroptosis activation. The ferritin complex is composed of heavy chain (H) and light chain (L) subunits. H- and L-ferritin in the absence (apo-) or presence (holo-) of combined iron were added 30 min prior to administration of RSL-3 (25 nM). After an 18 h incubation period, cell viability was analyzed. Data are presented as mean ± SD of biological triplicates (6 technical replicates, respectively). The normal distribution of the data was assessed using the Shapiro-Wilk test. Differences in ***A)*** (reference: 100 % iron), ***B)*** and ***D)*** were tested for significance by one-way ANOVA, followed by Dunnett's post hoc test. Differences in ***C)*** were tested by two-way ANOVA, followed by Dunnett's multiple comparison post hoc test. *p < 0.05 for comparison with untreated controls. Statistical analysis was performed using GraphPad Prism 5.0 (GraphPad Software, La Jolla, USA).

To estimate the extracellular iron supply limitation necessary to observe protection from ferroptosis activation, different iron concentrations were added during the differentiation period from day 2 to day 6. Total intracellular iron concentration (Fig. 8A) showed a maximal decrease when the extracellular iron supply was cut by > 90 %. To gain insight into the time required for adaptation of intracellular iron levels to changes in extracellular supply, iron-deprived (days 2–5) LUHMES cells were exposed on day 5 to a medium containing regular iron concentrations. Conversely, LUHMES cells differentiated in iron-containing medium until day 5 were exposed to iron-free medium on day 5 (Fig. 8B). The amount of intracellular iron was measured at different time points after the medium was changed. In both scenarios, a significant alteration in intracellular iron content was observed after approximately 12 h (Fig. 8B). Activation of RSL-3-dependent ferroptosis indicated the necessity of a more than 95 % (compared to standard supply = 100 %) reduction in extracellular iron for an effective protection of cell viability (>50 %) (Suppl. Fig. 7A). In a next step, ferroptosis was activated in LUHMES, differentiated in the presence of varying amounts of extracellular iron (up to 200 %) in the presence or absence of the iron chelator deferoxamine. A concentration-dependent decline in viability was observed with increasing iron availability. The presence of deferoxamine allowed a complete protection from ferroptosis. (Fig. 8C). To investigate the influence of a biologically relevant iron scavenging system, iron-free apoferritin H or iron-loaded ferritin H was added at different amounts together with RSL-3 to LUHMES differentiated in regular, iron-containing medium. Apoferritin H, but not iron-saturated ferritin-H was sufficient to almost completely protect cells from RSL-3-induced effects (Fig. 8 D and Suppl. Fig. 7B). These findings emphasize a modulation of intracellular iron levels by variations in extracellular iron supply and indicate a direct correlation between the amount of intracellular iron and the sensitivity of the cells toward ferroptosis induction.

### 3.7 Transcriptomics analysis

**Fig. 9:**
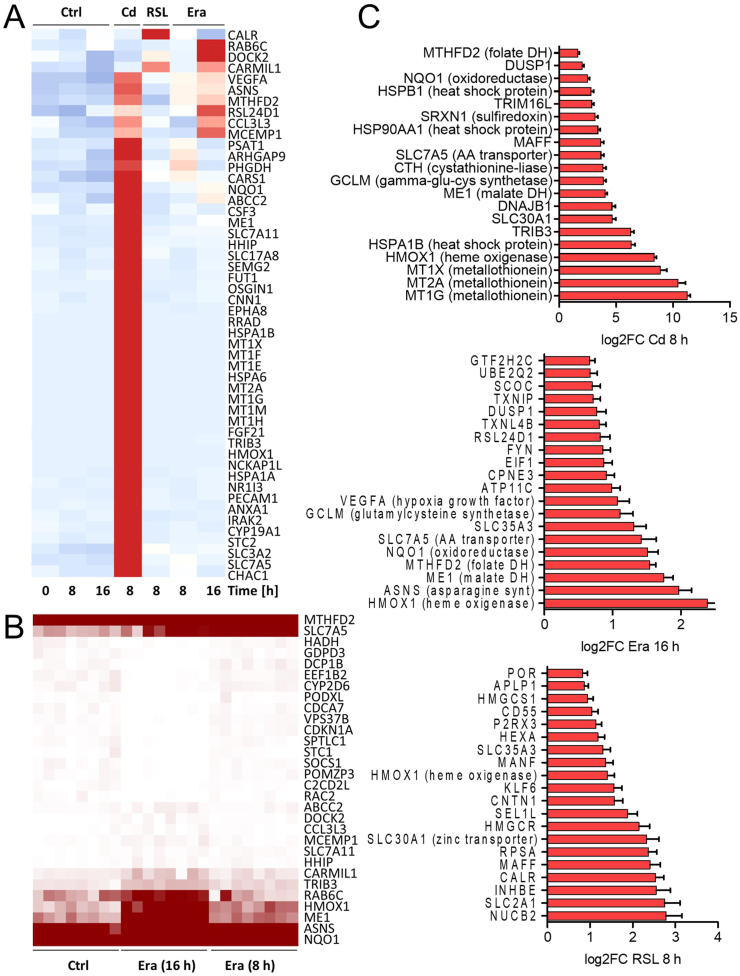
**Transcriptomics analysis**. Transcriptome data were obtained from day 6 LUHMES cells exposed to three different toxicants. The exposure times were chosen based on cytotoxicity data, such that the cells were still viable (>85 %) at lysis time (but close to cell death initiation). ***A)*** For each condition, average expression levels (in counts per million reads; CPM) were visualized in a heatmap of the top 50 upregulated genes. Genes were selected for heatmap display based on the results of the differential gene expression analysis for each toxicant to allow for a representative overview. The top 30 transcripts affected by cadmium (Cd) were selected, in addition to the top 15 each for 16 h erastin (Era) and 8 h RSL-3. Only genes with fold change ≥2, adjusted *p*-value ≤0.05 were included. The color scale represents z-scores, ranging from blue (−2) over white (0) to red (+2). ***B)*** The top 15 up- and downregulated genes by erastin (Era) at 16 h are visualized in a separate heatmap, where all replicates are shown (n = 8, as in A). The color scale ranges from 0 CPM (white) to 250 CPM (dark red); note that absolute values, not z-scores, are shown and that expression values > 250 cannot be distinguished (this information can be derived from C). ***C)*** For Era, RSL-3, and Cd, the top 20 upregulated genes are displayed as bar plots. (For interpretation of the references to color in this figure legend, the reader is referred to the Web version of this article.)

TempOSeq analysis was used to identify a potential ferroptosis transcriptome profile [50]. Cadmium (Cd^2+^), a well-established trigger of oxidative stress, served as a positive control. Cd^2+^-induced toxicity could not be prevented by ferroptosis-related interventions such as iron chelators (Suppl. Fig. 8A). Cd^2+^ triggered already after 8 h an extensive transcriptome response (more than 1000 differentially regulated genes; Fig. 9 and Suppl. Fig. 9 and 10). Many of the top differentially regulated genes were Nrf-2 targets and typically associated with oxidative stress, for example, *MT1G*, *PSAT1*, *ME1*, *HMOX1*, *CHAC1*, *NQO1*, and *GCLM*. Erastin triggered barely a response at 8 h, whereas RSL-3 triggered a clear but narrow response in terms of fold changes in regulated transcripts. A considerable response to erastin was observed after 16 h of treatment. The later time point was not sampled for RSL-3 because a considerable number of these cells had died at this time point. A synopsis showed that the two ferroptosis triggers induced a different and much-attenuated response compared with Cd^2+^. In addition, a unique transcriptomic ferroptosis response, as suggested in some reviews [[51], [52], [53], [54]], was not observed (Fig. 9A). Most of the differentially regulated genes were related to oxidative stress; however, the expression of many Cd^2+^-modulated genes was not altered at all by RSL-3 or erastin. One conclusion drawn from this overview is that ferroptosis cannot be characterized based on transcriptome changes in the LUHMES model.

To better understand the time dependence of transcriptome regulation, we carefully studied the response to erastin after 8 and 16 h. No surprising results were found, and most changes just became more pronounced over time (Fig. 9B and C and Suppl. Fig. 9 and 10). Compared with the up to 1000-fold changes in transcript levels induced by Cd^2+^, only a few genes were upregulated by erastin > 2-fold. These included the typical oxidative stress signature of *HMOX1*, *ME1*, *MTHFD2*, *NQO1*, *SLC7A5*, and *GCLM*. In addition, asparagine synthase (*ASNS*) was strongly regulated (also by Cd^2+^, but not by RSL-3). *ASNS* is upregulated by proteostatic stress and energy deficits (in reverse mode, ASNS converts asparagine into ATP). The overlap between erastin and RSL-3 responses was not large and included *DOCK2*, *CARMIL1* (requiring further investigation), and *HMOX1*, a well-established oxidative stress marker and Nrf-2 target in most cell types. Gene downregulation did not reveal any obvious patterns (no overrepresentation of pathways or gene ontology term groups; Suppl. Fig. 10).

In conclusion, the transcriptome data confirmed that oxidative stress plays a major role in ferroptosis and that the specific ferroptosis response clearly differs from a more general oxidative stress pattern mainly controlled by Nrf-2.

## 4 Discussion

A review of the relevant literature reveals a noticeable lack of consensus regarding the obligatory criteria for assessing ferroptosis. Parkinsons disease-associated neurodegeneration serves as a prime example of the involvement of ferroptosis due to elevated iron levels in affected brain areas [11,12]. Therefore, we opted for the LUHMES cell line, which enables the differentiation of human nigrostriatal dopamine neurons, as a model for Parkinson's disease research [[30], [31], [32]]. The observed variations in ferroptosis activation between proliferating and differentiated LUHMES cells, as well as with other cell models (Suppl. Fig. 4), highlight the challenges in comparing and integrating observations from different cell models or differentiation protocols. This challenge is particularly evident with widely used models such as SH-SY5Y or PC12 cells, which are employed in both undifferentiated and differentiated states. The situation is further complicated by diverse differentiation protocols in the literature, hindering direct result comparisons [[55], [56], [57]].

To address these limitations, a two-stage LUHMES differentiation protocol has been previously established [[30], [31], [32]], emphasizing factors such as cell number, differentiation periods, number of medium changes, and medium constituents. Strict adherence to this protocol has been shown to ensure interlaboratory consistency of experimental observations [[37], [38], [39], [40]]. The two-step differentiation protocol halts proliferation of non-synchronized cells (days 0–2), enabling reproducible seeding of non-proliferating cells. The significance of LUHMES cell number has been underscored in prior work involving the parkinsonian toxicant MPP^+^ [30,31,58] and is particularly relevant for erastin and RSL-3 application. Both compounds exhibit optimal ferroptosis activation within narrow concentration ranges (erastin: 1.0–1.5 μM; RSL-3: 15–25 nM), as demonstrated in this study and corroborated by independent literature [28,29].

During our work, we noted that both erastin and RSL-3 in aqueous buffers behave more like suspensions, susceptible to gravitational influence. Therefore, considering a dosage rather than a concentration is crucial not only for compound addition to cell cultures but also for experimental design and data interpretation. Fig. 5B illustrates a concentration- and cell-density-dependent ferroptosis activation by RSL-3. Conversely, depicting the same dataset as exposure of an individual cell to different doses clearly indicates ferroptotic cell death activation at the same dose per cell over the range of cell densities and RSL-3 concentrations tested. This example underscores the importance of the cell-to-dose ratio for ferroptosis activation.

In our study, ferroptosis was induced using the cystine/glutamate antiporter inhibitor erastin or RSL-3, the latter considered an exclusive inhibitor of GPx4 activity [59,60]. However, a recent report challenged this notion, suggesting RSL-3 as inhibitor of thioredoxin reductase 1 rather than GPx4 [61]. Another report confirmed the absence of efficient inhibition of recombinant GPx4, however also indicated that this effect could be reversed by the addition of cell cytosol, or the adaptor protein 14-3-3 epsilon [62]. The contribution of thioredoxin reductase 1 or GPx4 inhibition by RSL-3 in LUHMES remains unclear at present. Nevertheless, in differentiated LUHMES, features associated with ferroptosis activation and protection from ferroptosis were observed for both erastin and RSL-3 treatments, justifying RSL-3's consideration as a ferroptosis activator.

Our findings (Fig. 3, Fig. 7, Fig. 8) demonstrate a significant impact of iron supply through media on ferroptosis activation. Holo-transferrin in Advanced DMEM/F12 medium represents a predominant iron source. The degree of iron saturation of commercially available holo-transferrin can vary considerably, necessitating careful consideration in experimental design and result interpretation. LUHMES maintenance also involves N2 supplement, another potent iron source, while FCS is commonly used for other cell models. Both N2 and FCS, along with their amount, timing of addition, and duration of exposure, significantly influence ferroptosis activation and warrant careful consideration in the design of an experiment. Similar to the limitation imposed by iron supply (Fig. 7), iron chelator application efficiently protects against ferroptotic cell death (Fig. 3). Deferoxamine, for instance, reportedly shields all coordination sites of iron, thus preventing hydroxyl radical formation by complexed iron [63]. However, its cellular uptake in LUHMES remains uncertain, with conflicting literature reporting either lack of uptake or endocytic pathway uptake followed by lysosomal sequestration [64,65]. Considering the rapid exchange between extracellular and intracellular iron, extracellular iron chelators may lower extracellular iron levels, creating a new concentration gradient that facilitates intracellular iron efflux, thereby protecting cells from iron-catalyzed radical formation.

Additionally, pharmacological interventions at diverse molecular targets allow almost complete protection from erastin- or RSL-3-dependent ferroptosis (Fig. 4). This raises questions about how single-site interference enables efficient protection from cell death. Ferroptosis activation is expected at a defined tipping point where lipid peroxidation levels exceed a threshold. It is conceivable that erastin or RSL-3 exposure triggers multiple adverse mechanisms simultaneously. Near tipping points, interventions at individual ferroptosis-contributing mechanisms may suffice to delay or prevent ferroptotic cell death. Characterizing such tipping points in specific cell types could inform pharmacological strategies targeting ferroptosis activation in cancer cells while preserving other cell types.

## 5 Conclusions and outlook

Utilizing *in vitro* models for the prediction of molecular events *in vivo* is obviously a difficult endeavor. With respect to the LUHMES/ferroptosis model, the following aspects should be taken into consideration:

(i) in the absence of a broadly accepted marker, ferroptosis is largely defined by the sensitivity of a cell towards defined inducers or inhibitors. This can be realized with high accuracy in a homogenous cell model, but with more difficulties and limitations *in vivo.*
(ii) different types of cell death (ferroptosis, apoptosis, oxeiptosis) often appear to be interrelated. Detailed investigations require single cell analysis of such death processes, which is technically more feasible in an *in vitro* model than *in vivo*.
(iii) rodents are regularly used *in vivo* models in ferroptosis research. For pharmacological investigations, differences between mouse and human proteins, such as in FSL-1 are known and influence the binding of e.g. the FSL-1 inhibitor iFSL-1 [66,67].
(iv) ferroptosis is dependent on the cellular redox state and on various cellular pools of antioxidants and prooxidants. These factors might be strongly biased by the *in vitro* experimental conditions. However, such factors may also be biased in experimental animal models, e.g. due to standard chows, or as consequence of differences between rodents and humans. (*v*) ferroptosis is highly dependent on the expression pattern of proteins such as GPx4, FSP-1, or DHODH. The individual expression pattern in an *in vitro* model hence may represent only a small portion of cell types *in vivo.*

**Fig. 10:**
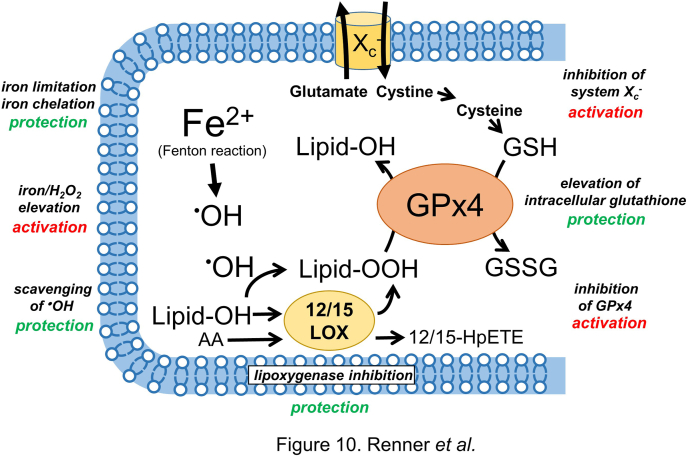
**Representation of cardinal features of ferroptosis by the LUHMES model**. Ferroptosis is characterized by an iron-catalyzed formation of hydroxyl radicals (^•^OH) and their oxidation of polyunsaturated fatty acids resulting in the formation of lipid hydroperoxides (lipid-OOH). ***Activation*** of ferroptosis in differentiated LUHMES can be achieved by an inhibition of the cystine/glutamate antiporter system X_c_^−^, by an inhibition of glutathione peroxidase 4 (GPx4), or by a concomitant elevation of iron and hydroperoxides. ***Protection*** from ferroptosis can be achieved by an experimental limitation of cellular iron, by scavenging of hydroxyl radicals, by an inhibition of cellular lipoxygenases, or by an experimental elevation of intracellular glutathione.

These considerations illustrate the limitations of *in vitro* models but also highlight research areas in which thoroughly characterized *in vitro* models can make a valuable contribution to current ferroptosis research (Fig. 10).

## CRediT authorship contribution statement

**Nadine Renner:** Investigation, Formal analysis, Methodology, Visualization, Writing – original draft. **Franziska Schöb:** Investigation, Methodology, Formal analysis. **Regina Pape:** Investigation, Formal analysis. **Ilinca Suciu:** Data curation, Formal analysis, Software. **Anna-Sophie Spreng:** Formal analysis, Methodology, Investigation. **Anna-Katharina Ückert:** Formal analysis, Methodology, Investigation. **Eike Cöllen:** Formal analysis, Investigation. **Federica Bovio:** Formal analysis, Investigation. **Bruno Chilian:** Investigation, Methodology. **Johannes Bauer:** Formal analysis, Investigation. **Stefan Röpcke:** Data curation, Formal analysis, Resources. **Jörg Bergemann:** Formal analysis, Investigation. **Marcel Leist:** Conceptualization, Funding acquisition, Investigation, Project administration. **Stefan Schildknecht:** Investigation, Methodology, Project administration, Resources, Supervision, Writing – original draft, Writing – review & editing, Conceptualization, Formal analysis, Funding acquisition.

## Declaration of competing interest

The authors declare that they have no known competing financial interests or personal relationships that could have appeared to influence the work reported in this paper.

## Data Availability

Data will be made available on request.
