# Supplementary material for: Modeling ferroptosis in human dopaminergic neurons: Pitfalls and opportunities for neurodegeneration research
Source: Redox Biol. 2024 Apr 24;73:103165. doi: 10.1016/j.redox.2024.103165 (PMC11070765; doi:10.1016/j.redox.2024.103165)
Supplement: Multimedia component 1 [file mmc1.pptx]

## Slide 1
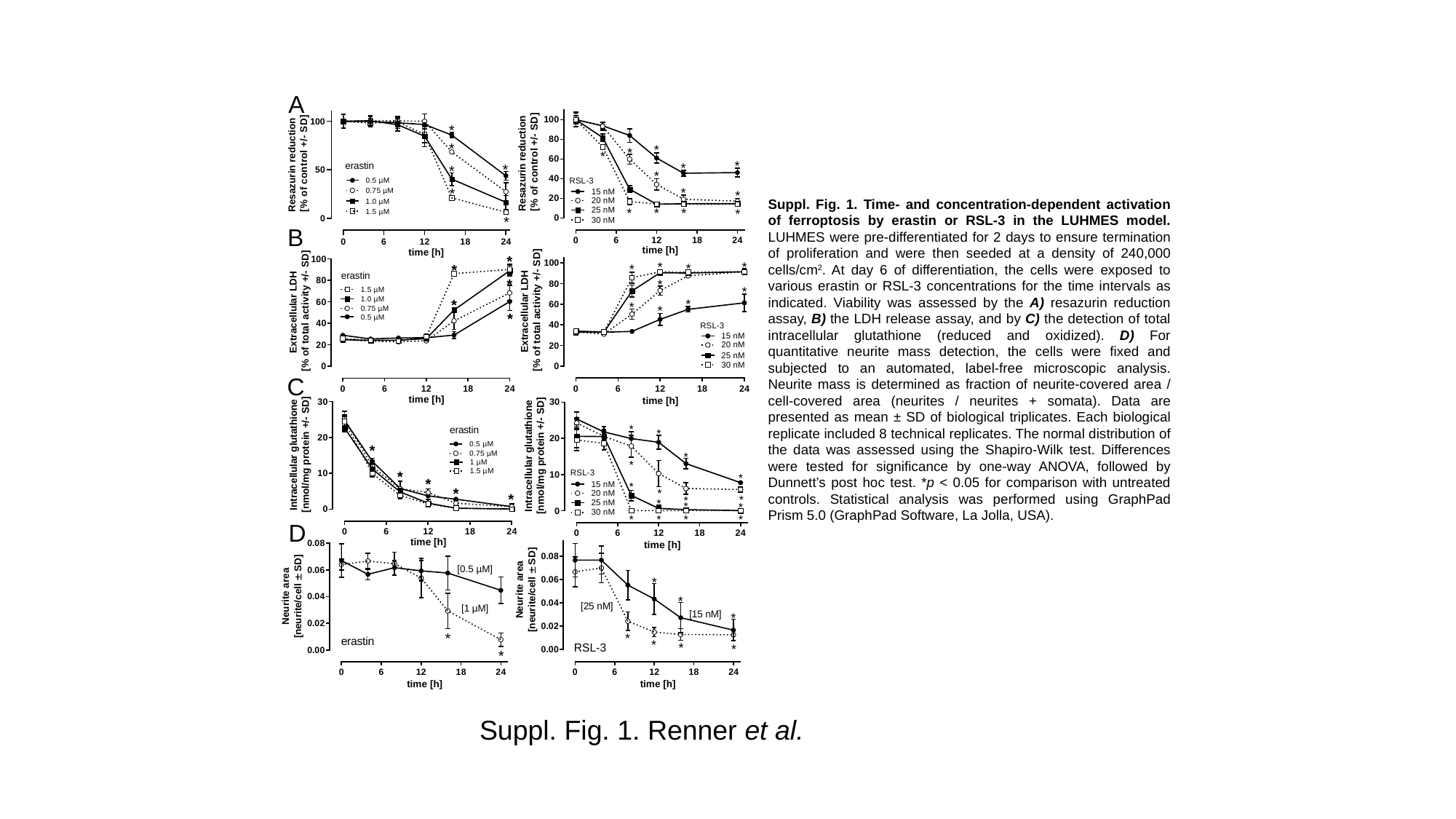

A
Suppl. Fig. 1. Time- and concentration-dependent activation of ferroptosis by erastin or RSL-3 in the LUHMES model. LUHMES were pre-differentiated for 2 days to ensure termination of proliferation and were then seeded at a density of 240,000 cells/cm2. At day 6 of differentiation, the cells were exposed to various erastin or RSL-3 concentrations for the time intervals as indicated. Viability was assessed by the A) resazurin reduction assay, B) the LDH release assay, and by C) the detection of total intracellular glutathione (reduced and oxidized). D) For quantitative neurite mass detection, the cells were fixed and subjected to an automated, label-free microscopic analysis. Neurite mass is determined as fraction of neurite-covered area / cell-covered area (neurites / neurites + somata). Data are presented as mean ± SD of biological triplicates. Each biological replicate included 8 technical replicates. The normal distribution of the data was assessed using the Shapiro-Wilk test. Differences were tested for significance by one-way ANOVA, followed by Dunnett’s post hoc test. *p < 0.05 for comparison with untreated controls. Statistical analysis was performed using GraphPad Prism 5.0 (GraphPad Software, La Jolla, USA).
B
C
D
Suppl. Fig. 1. Renner et al.

## Slide 2
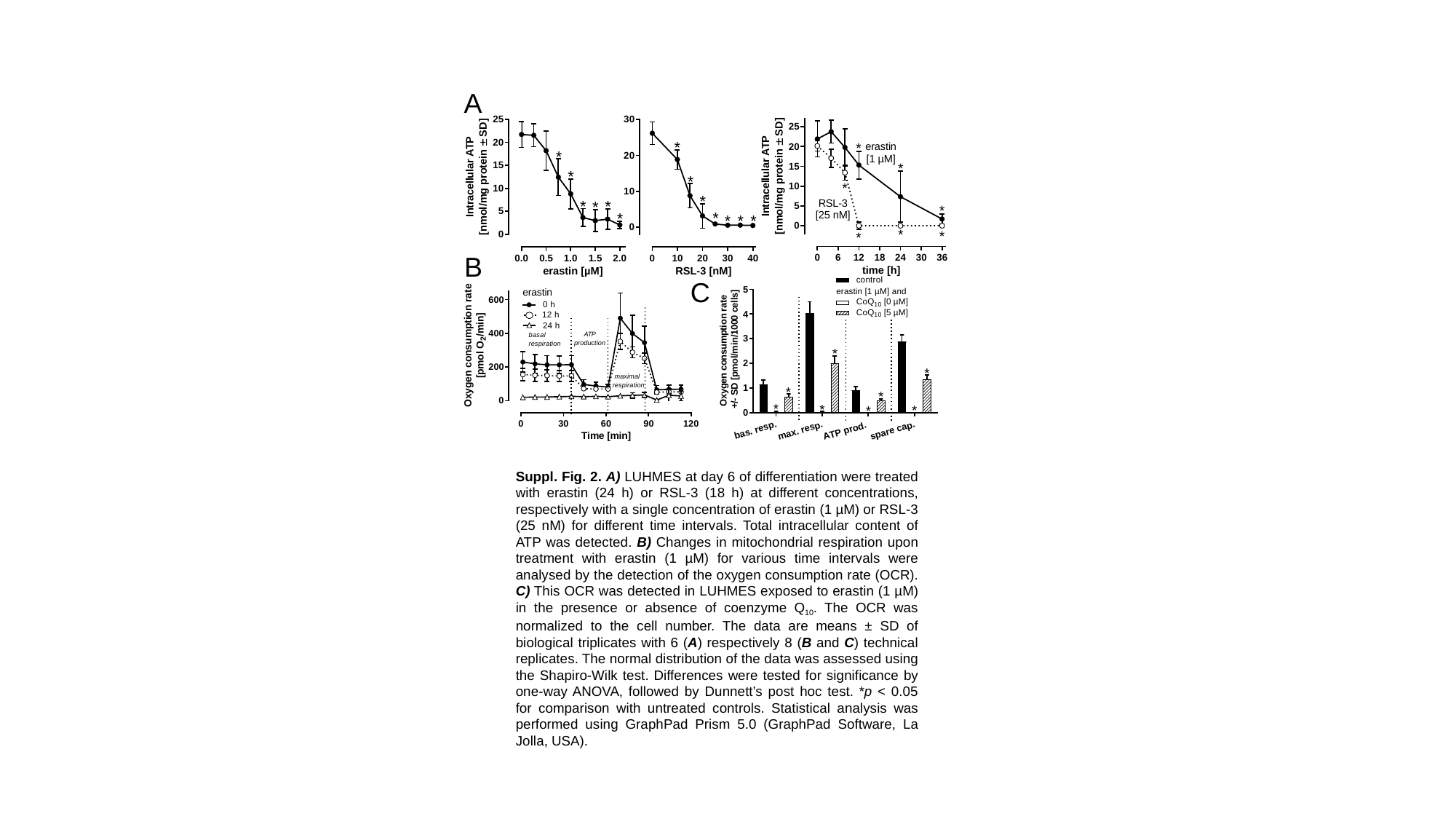

A
B
C
Suppl. Fig. 2. A) LUHMES at day 6 of differentiation were treated with erastin (24 h) or RSL-3 (18 h) at different concentrations, respectively with a single concentration of erastin (1 µM) or RSL-3 (25 nM) for different time intervals. Total intracellular content of ATP was detected. B) Changes in mitochondrial respiration upon treatment with erastin (1 µM) for various time intervals were analysed by the detection of the oxygen consumption rate (OCR). C) This OCR was detected in LUHMES exposed to erastin (1 µM) in the presence or absence of coenzyme Q10. The OCR was normalized to the cell number. The data are means ± SD of biological triplicates with 6 (A) respectively 8 (B and C) technical replicates. The normal distribution of the data was assessed using the Shapiro-Wilk test. Differences were tested for significance by one-way ANOVA, followed by Dunnett’s post hoc test. *p < 0.05 for comparison with untreated controls. Statistical analysis was performed using GraphPad Prism 5.0 (GraphPad Software, La Jolla, USA).
Suppl. Fig. 2. Renner et al.

## Slide 3
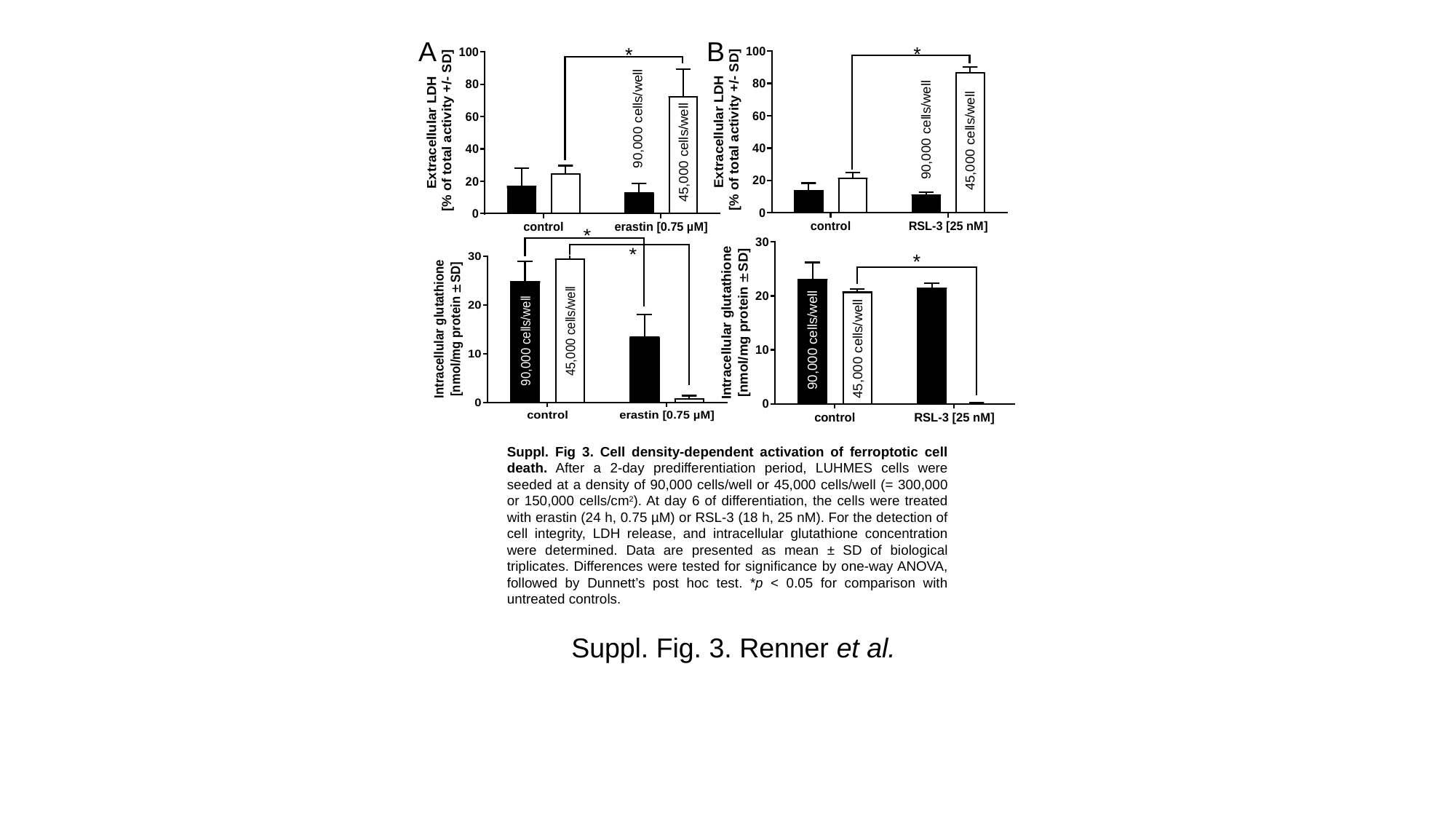

B
A
Suppl. Fig 3. Cell density-dependent activation of ferroptotic cell death. After a 2-day predifferentiation period, LUHMES cells were seeded at a density of 90,000 cells/well or 45,000 cells/well (= 300,000 or 150,000 cells/cm2). At day 6 of differentiation, the cells were treated with erastin (24 h, 0.75 µM) or RSL-3 (18 h, 25 nM). For the detection of cell integrity, LDH release, and intracellular glutathione concentration were determined. Data are presented as mean ± SD of biological triplicates. Differences were tested for significance by one-way ANOVA, followed by Dunnett’s post hoc test. *p < 0.05 for comparison with untreated controls.
Suppl. Fig. 3. Renner et al.

## Slide 4
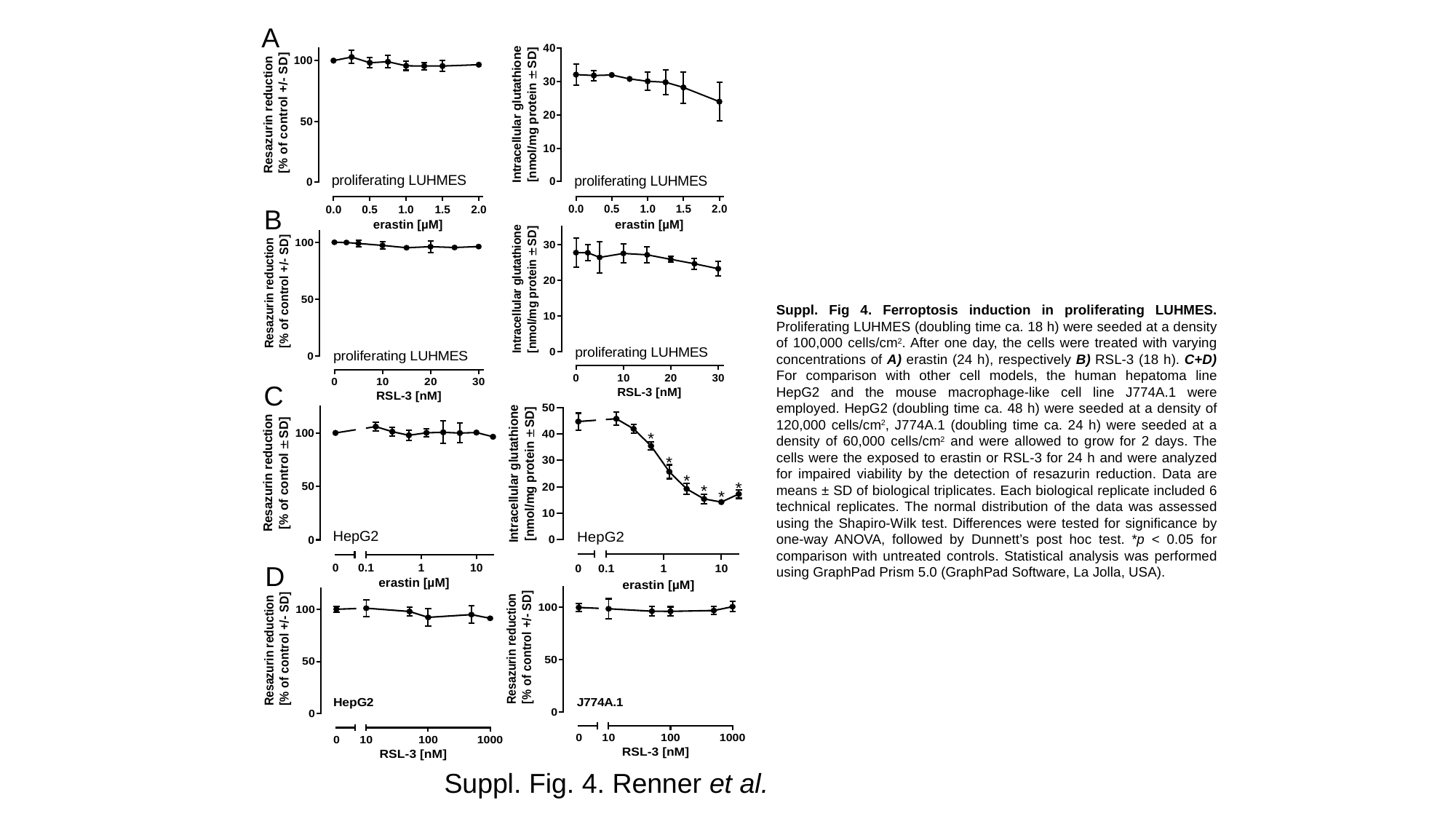

A
B
Suppl. Fig 4. Ferroptosis induction in proliferating LUHMES. Proliferating LUHMES (doubling time ca. 18 h) were seeded at a density of 100,000 cells/cm2. After one day, the cells were treated with varying concentrations of A) erastin (24 h), respectively B) RSL-3 (18 h). C+D) For comparison with other cell models, the human hepatoma line HepG2 and the mouse macrophage-like cell line J774A.1 were employed. HepG2 (doubling time ca. 48 h) were seeded at a density of 120,000 cells/cm2, J774A.1 (doubling time ca. 24 h) were seeded at a density of 60,000 cells/cm2 and were allowed to grow for 2 days. The cells were the exposed to erastin or RSL-3 for 24 h and were analyzed for impaired viability by the detection of resazurin reduction. Data are means ± SD of biological triplicates. Each biological replicate included 6 technical replicates. The normal distribution of the data was assessed using the Shapiro-Wilk test. Differences were tested for significance by one-way ANOVA, followed by Dunnett’s post hoc test. *p < 0.05 for comparison with untreated controls. Statistical analysis was performed using GraphPad Prism 5.0 (GraphPad Software, La Jolla, USA).
C
D
Suppl. Fig. 4. Renner et al.

## Slide 5
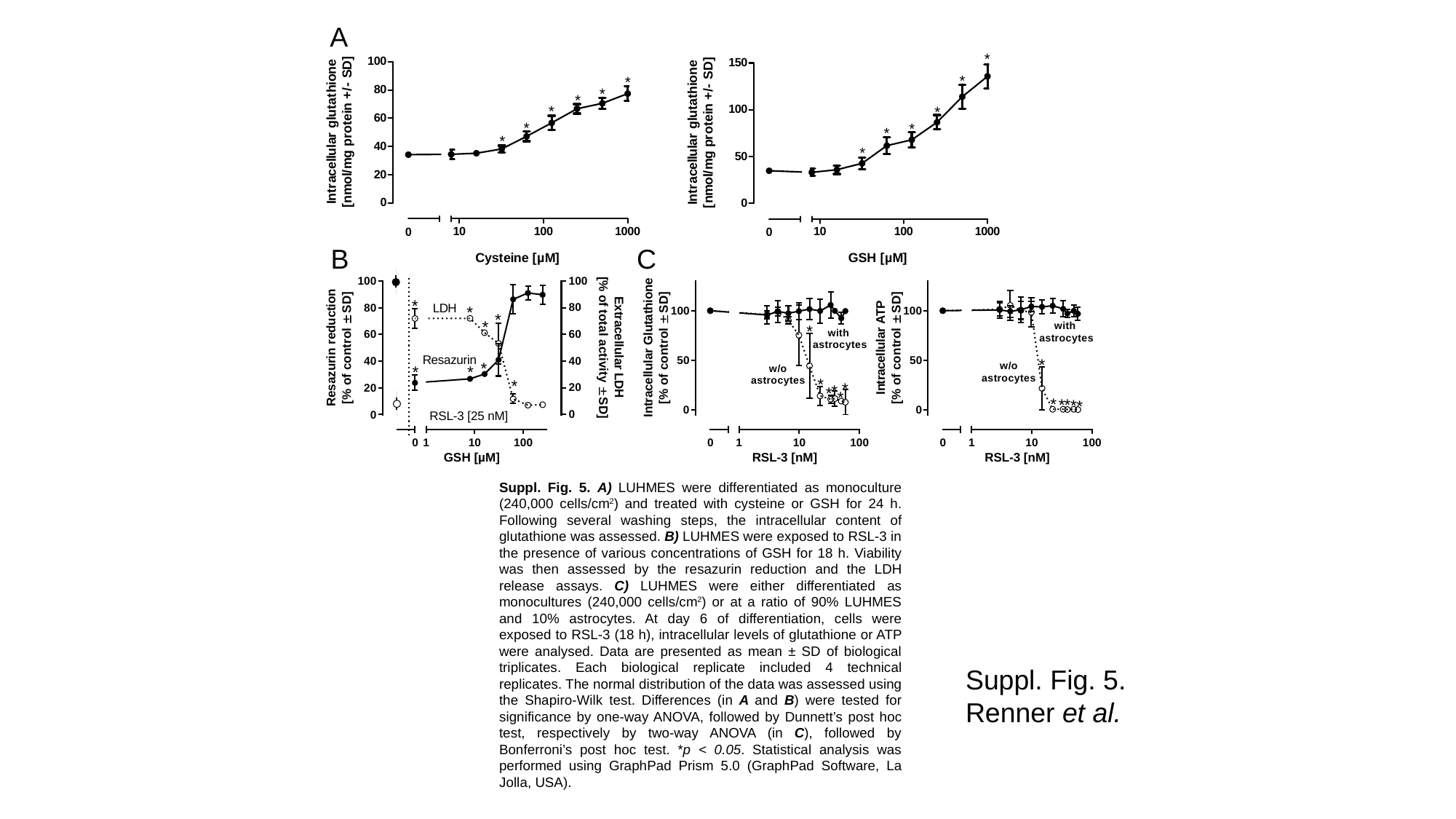

A
B
C
Suppl. Fig. 5. A) LUHMES were differentiated as monoculture (240,000 cells/cm2) and treated with cysteine or GSH for 24 h. Following several washing steps, the intracellular content of glutathione was assessed. B) LUHMES were exposed to RSL-3 in the presence of various concentrations of GSH for 18 h. Viability was then assessed by the resazurin reduction and the LDH release assays. C) LUHMES were either differentiated as monocultures (240,000 cells/cm2) or at a ratio of 90% LUHMES and 10% astrocytes. At day 6 of differentiation, cells were exposed to RSL-3 (18 h), intracellular levels of glutathione or ATP were analysed. Data are presented as mean ± SD of biological triplicates. Each biological replicate included 4 technical replicates. The normal distribution of the data was assessed using the Shapiro-Wilk test. Differences (in A and B) were tested for significance by one-way ANOVA, followed by Dunnett’s post hoc test, respectively by two-way ANOVA (in C), followed by Bonferroni’s post hoc test. *p < 0.05. Statistical analysis was performed using GraphPad Prism 5.0 (GraphPad Software, La Jolla, USA).
Suppl. Fig. 5.
Renner et al.

## Slide 6
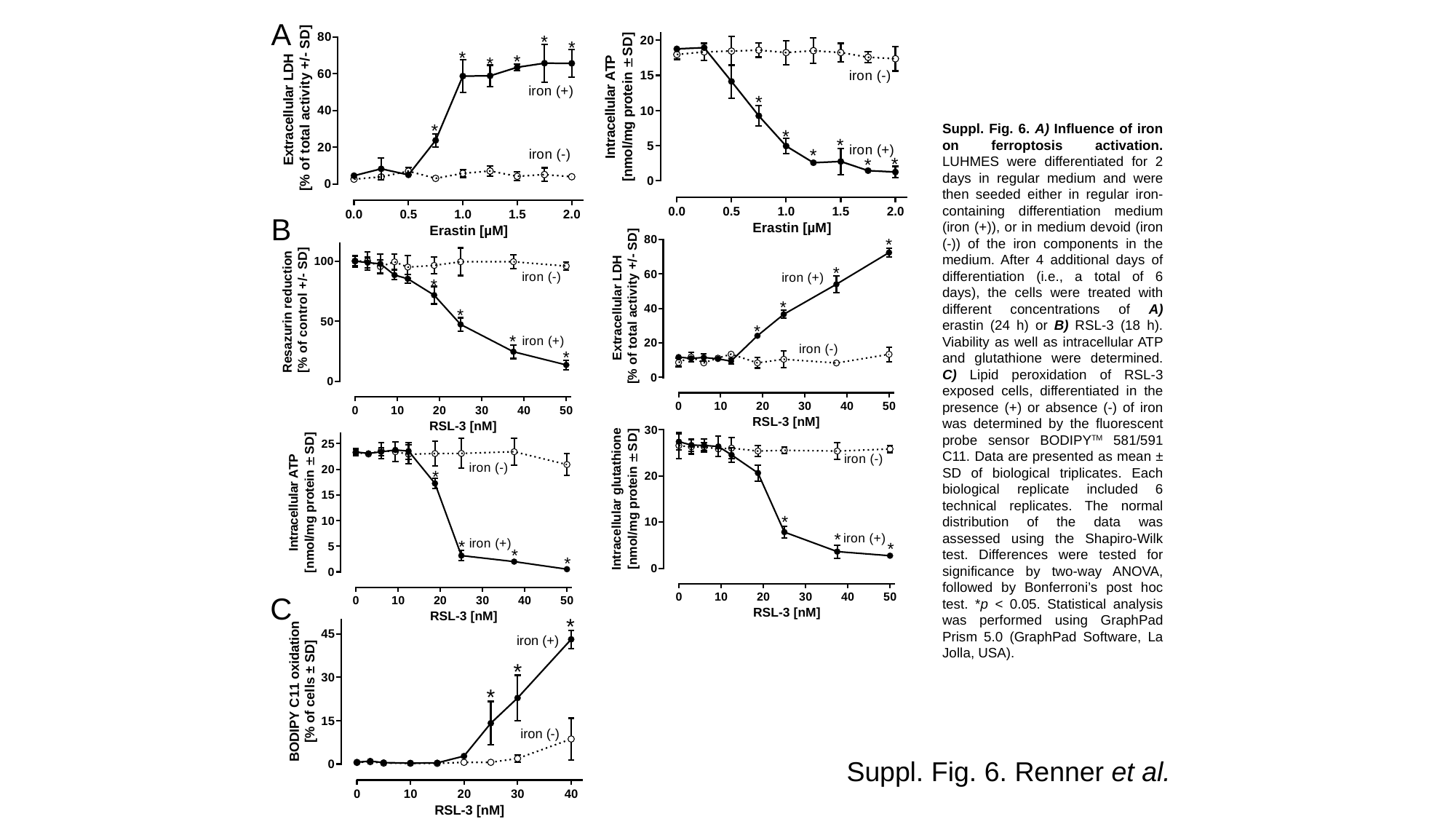

A
Suppl. Fig. 6. A) Influence of iron on ferroptosis activation. LUHMES were differentiated for 2 days in regular medium and were then seeded either in regular iron-containing differentiation medium (iron (+)), or in medium devoid (iron (-)) of the iron components in the medium. After 4 additional days of differentiation (i.e., a total of 6 days), the cells were treated with different concentrations of A) erastin (24 h) or B) RSL-3 (18 h). Viability as well as intracellular ATP and glutathione were determined. C) Lipid peroxidation of RSL-3 exposed cells, differentiated in the presence (+) or absence (-) of iron was determined by the fluorescent probe sensor BODIPYTM 581/591 C11. Data are presented as mean ± SD of biological triplicates. Each biological replicate included 6 technical replicates. The normal distribution of the data was assessed using the Shapiro-Wilk test. Differences were tested for significance by two-way ANOVA, followed by Bonferroni’s post hoc test. *p < 0.05. Statistical analysis was performed using GraphPad Prism 5.0 (GraphPad Software, La Jolla, USA).
B
C
Suppl. Fig. 6. Renner et al.

## Slide 7
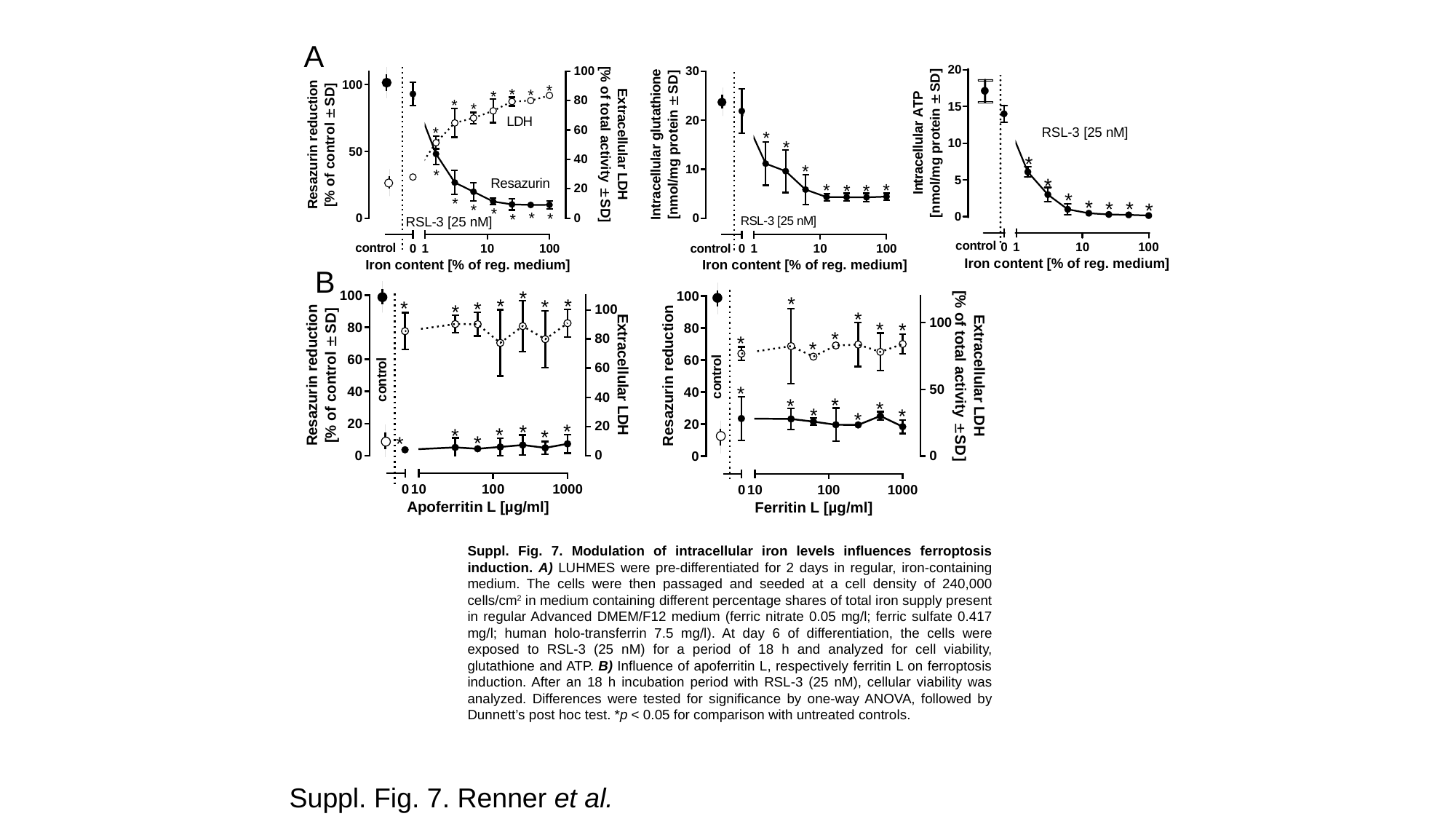

A
B
Suppl. Fig. 7. Modulation of intracellular iron levels influences ferroptosis induction. A) LUHMES were pre-differentiated for 2 days in regular, iron-containing medium. The cells were then passaged and seeded at a cell density of 240,000 cells/cm2 in medium containing different percentage shares of total iron supply present in regular Advanced DMEM/F12 medium (ferric nitrate 0.05 mg/l; ferric sulfate 0.417 mg/l; human holo-transferrin 7.5 mg/l). At day 6 of differentiation, the cells were exposed to RSL-3 (25 nM) for a period of 18 h and analyzed for cell viability, glutathione and ATP. B) Influence of apoferritin L, respectively ferritin L on ferroptosis induction. After an 18 h incubation period with RSL-3 (25 nM), cellular viability was analyzed. Differences were tested for significance by one-way ANOVA, followed by Dunnett’s post hoc test. *p < 0.05 for comparison with untreated controls.
Suppl. Fig. 7. Renner et al.

## Slide 8
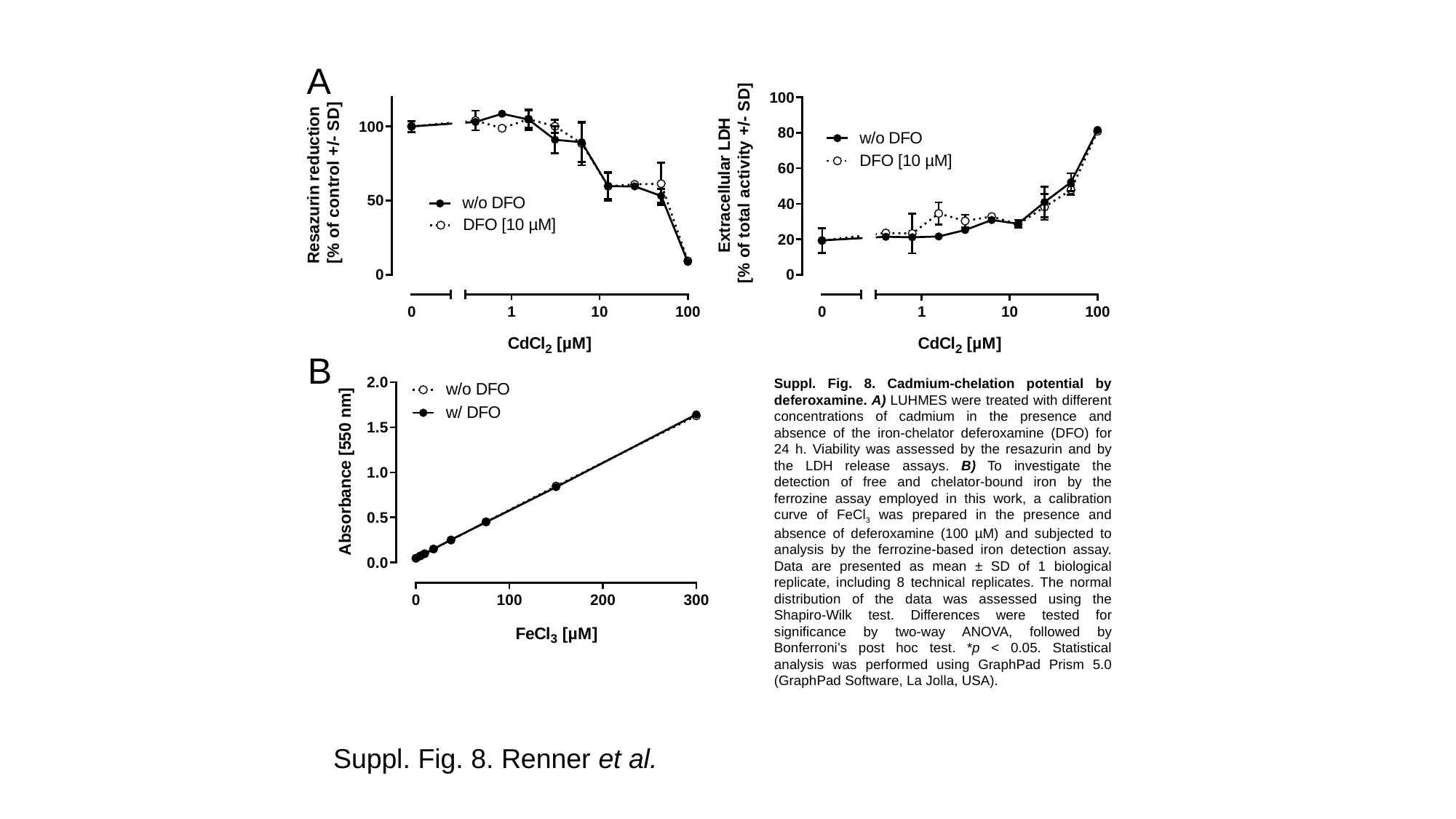

A
B
Suppl. Fig. 8. Cadmium-chelation potential by deferoxamine. A) LUHMES were treated with different concentrations of cadmium in the presence and absence of the iron-chelator deferoxamine (DFO) for 24 h. Viability was assessed by the resazurin and by the LDH release assays. B) To investigate the detection of free and chelator-bound iron by the ferrozine assay employed in this work, a calibration curve of FeCl3 was prepared in the presence and absence of deferoxamine (100 µM) and subjected to analysis by the ferrozine-based iron detection assay. Data are presented as mean ± SD of 1 biological replicate, including 8 technical replicates. The normal distribution of the data was assessed using the Shapiro-Wilk test. Differences were tested for significance by two-way ANOVA, followed by Bonferroni’s post hoc test. *p < 0.05. Statistical analysis was performed using GraphPad Prism 5.0 (GraphPad Software, La Jolla, USA).
Suppl. Fig. 8. Renner et al.

## Slide 9
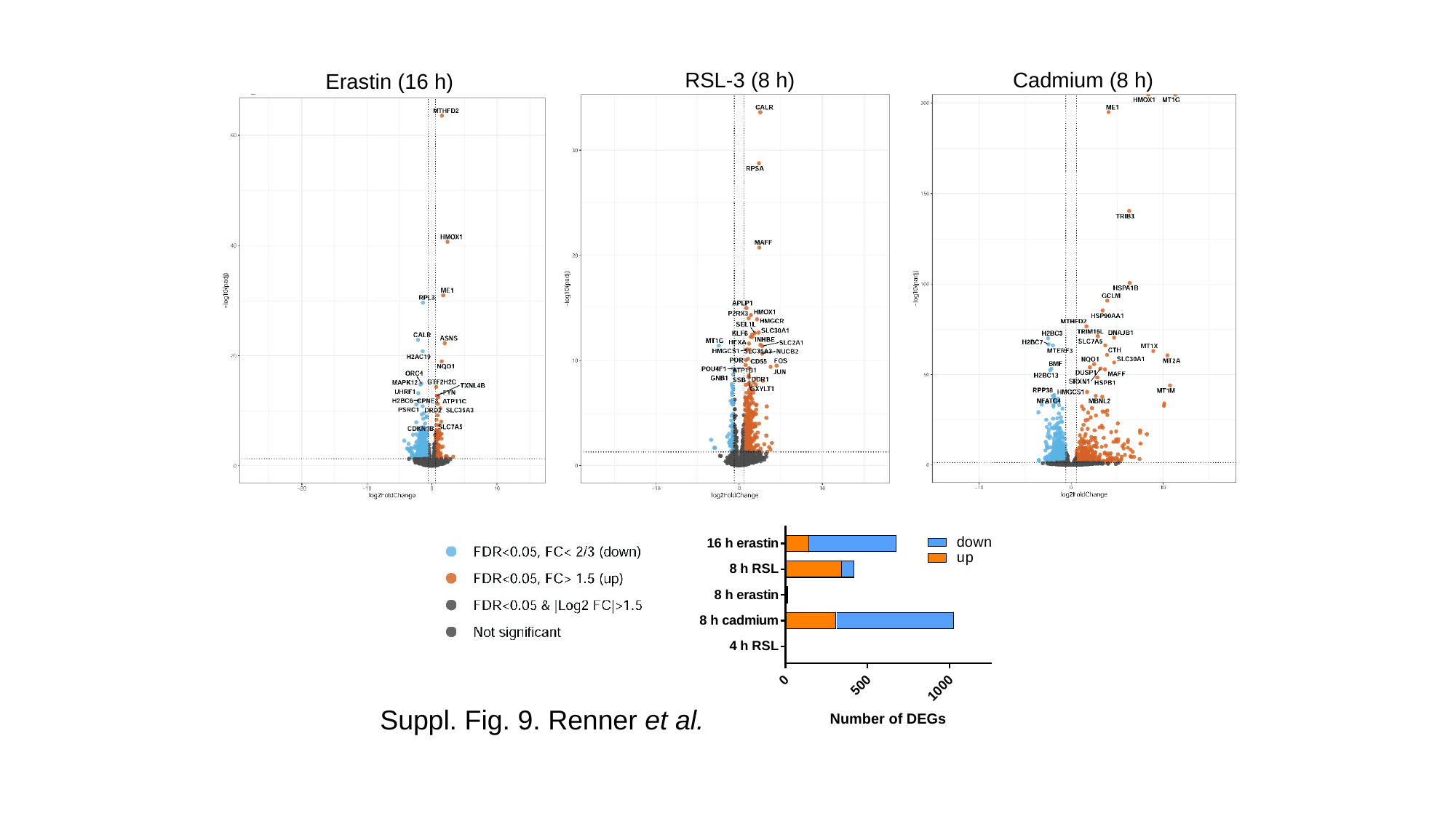

RSL-3 (8 h)
Cadmium (8 h)
Erastin (16 h)
Suppl. Fig. 9. Renner et al.

## Slide 10
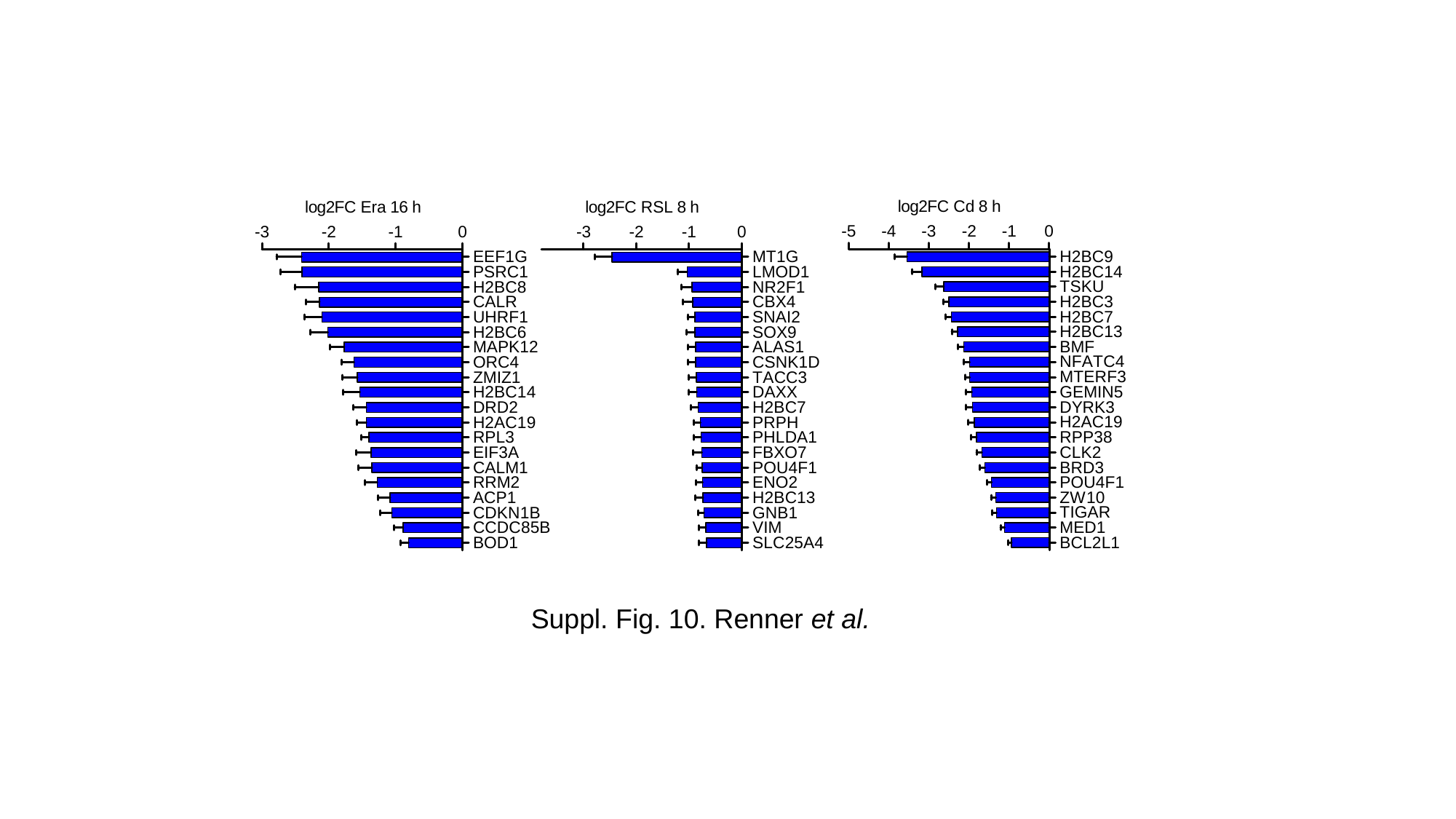

Suppl. Fig. 10. Renner et al.
